# Environmental exposure to lead and cadmium are associated with triglyceride glucose index

**DOI:** 10.1038/s41598-024-52994-5

**Published:** 2024-01-30

**Authors:** Taiyue Jin, Eun Young Park, Byungmi Kim, Jin-Kyoung Oh

**Affiliations:** 1 Division of Cancer Prevention, National Cancer Control Institute, National Cancer Center, Goyang-si, 10408 Gyeonggi-do Korea; 2 Department of Preventive Medicine, Korea University College of Medicine, 73 Goryeodae-ro, Seongbuk-gu, Seoul, 02841 Korea; 3 Department of Cancer Control and Population Health, Graduate School of Cancer Science and Policy, National Cancer Center, Goyang-si, 10408 Gyeonggi-do Korea

**Keywords:** Predictive markers, Type 2 diabetes

## Abstract

The triglyceride glucose (TyG) index was suggested as a novel reliable surrogate marker for insulin resistance and related cardiovascular-metabolic diseases. We aimed to evaluate the association between the TyG index and environmental exposure to lead (Pb), mercury (Hg), and cadmium (Cd). A total of 9645 adults who enrolled in the Korea National Health and Nutrition Examination Survey in 2005, 2008–2013, and 2016 were included. Fasting plasma glucose and triglyceride levels were used to calculate the TyG index. Multivariate logistic regression model was used to estimate odds ratios (ORs) and 95% confidence intervals (CIs). We noted an increasing trend in the TyG index with increment of blood Pb and Cd concentrations. Participants in the highest quartile of blood Pb and Cd concentrations had higher TyG index values than those in the lowest quartile, with ORs (95% CIs) of 1.32 (1.07–1.63) and 1.29 (1.04–1.59) for Pb and Cd, respectively. Strong associations between blood Pb and Cd concentrations and the TyG index were found in men. Blood Hg concentrations did not show a significant association with the TyG index. Our study suggests that public health strategies for cardiovascular-metabolic disorder prevention should be directed toward individuals exposed to priority heavy metals.

## Introduction

Lead (Pb), mercury (Hg), and cadmium (Cd) are heavy metals found naturally in the earth’s crust and seawater^1^. Environmental exposure to these elements occurs mainly through industrial, agricultural, anthropogenic, and cosmetic activities^2^. Given their high degree of toxicity, bioaccumulation, and persistence in the body, environmental monitoring of Pb, Hg, and Cd—considered priority heavy metals by the United States (US) Environmental Protection Agency (EPA)^3^—is of great significance to public health. These heavy metals have been reported to be associated with cardiovascular-metabolic disorders, such as cardiovascular disease, type 2 diabetes mellitus (T2DM), hypertension, and obesity^4–9^.

Recently, the triglyceride glucose (TyG) index, a product of fasting glucose and triglyceride (TG) levels, was proposed as a more cost-effective and accessible surrogate marker for insulin resistance (IR). Referring to the hyperinsulinemic-euglycemic clamp (HEC) test, the TyG index might achieve better detection performance than homeostatic model assessment of insulin resistance (HOMA-IR)^10^. Some prospective and cross-sectional studies have reported that use of the TyG index as surrogate marker for IR^11,12^, cardiovascular diseases (CVDs)^11^, and metabolic syndrome (MetS)^13^. Optimal cut-off points of the TyG index for IR identification were determined in two Asian populations: Chinese and Japanese^11,12^. In an Iranian cohort study, a corresponding cut-off point for 10-year incident CVD and coronary heart disease was reported^14^. Moreover, in Korean population, a cut-off point of the TyG index for predicting incident MetS with a sensitivity of 73% and a specificity of 82% was proposed^13^. Therefore, the TyG index might be a useful tool for screening the risk of IR and related cardiovascular-metabolic diseases in healthy populations, especially, when insulin measurement is not feasible^15^.

In this context, to our knowledge, there was no investigation evaluate the association between heavy metals and the TyG index. Hence, in this study, we aimed to confirm whether environmental exposure to Pb, Hg, and Cd was associated with the TyG index in Korean general population by adopting several the cut-off points proposed in the extant literature.

## Results

### Baseline characteristics

The TyG index ranged from 5.89 to 12.73, and the geometric mean (GM) (95% confidence interval [CI]) was 8.43 (8.41–8.44). Table 1 shows the baseline characteristics of the study participants according to the TyG index quartiles. Participants in the highest quartile of the TyG index were more likely to be older, men, obese, heavy drinkers, current smokers, less educated, and inactive and had a higher grain and vegetable consumption compared with those in the lowest quartile. Moreover, the highest quartile of the TyG index had a higher proportion of postmenopausal women than the lowest quartile.

**Table 1:** Baseline characteristics of the study participants according to the TyG index.

	Overall (n = 9645)	TyG index				*p*^a^
		Quartile 1 (5.89–7.98) (n = 2412)	Quartile 2 (7.99–8.39) (n = 2416)	Quartile 3 (8.40–8.83) (n = 2405)	Quartile 4 (8.84–12.73) (n = 2412)	
Age, mean ± SE, years	40.1 ± 0.2	35.2 ± 0.3	39.0 ± 0.3	42.2 ± 0.3	44.2 ± 0.3	< 0.001
Sex						
Men, n (%)	4314 (44.7)	652 (27.0)	901 (37.3)	1205 (50.1)	1556 (64.5)	< 0.001
Women, n (%)	5331 (55.3)	1760 (73.0)	1515 (62.7)	1200 (49.9)	856 (35.5)	
Survey year						
2005, n (%)	1333 (13.8)	300 (12.4)	351 (14.5)	335 (13.9)	347 (14.4)	0.308
2008, n (%)	1234 (12.8)	307 (12.7)	308 (12.7)	314 (13.1)	305 (12.6)	
2009, n (%)	1259 (13.1)	321 (13.3)	289 (12.0)	326 (13.6)	323 (13.4)	
2010, n (%)	1185 (12.3)	318 (13.2)	323 (13.4)	276 (11.5)	268 (11.1)	
2011, n (%)	1261 (13.1)	321 (13.3)	307 (12.7)	325 (13.5)	308 (12.8)	
2012, n (%)	1148 (11.9)	297 (12.3)	287 (11.9)	275 (11.4)	289 (12.0)	
2013, n (%)	1114 (11.6)	287 (11.9)	280 (11.6)	258 (10.7)	289 (12.0)	
2016, n (%)	1111 (11.5)	261 (10.8)	271 (11.2)	296 (12.3)	283 (11.7)	
BMI, mean ± SE, kg/m^2^	23.4 ± 0	21.8 ± 0.1	22.6 ± 0.1	23.9 ± 0.1	25.2 ± 0.1	< 0.001
Normal weight, n (%)	4748 (49.2)	1721 (71.4)	1418 (58.7)	1003 (41.7)	606 (25.1)	< 0.001
Overweight, n (%)	2161 (22.4)	402 (16.7)	526 (21.8)	617 (25.7)	616 (25.5)	
Obesity, n (%)	2736 (28.4)	289 (12.0)	472 (19.5)	785 (32.6)	1190 (49.3)	
Alcohol consumption						
Non-drinkers, n (%)	1865 (19.3)	466 (19.3)	500 (20.7)	503 (20.9)	396 (16.4)	< 0.001
< 1 drink/day, n (%)	5367 (55.6)	1580 (65.5)	1422 (58.9)	1294 (53.8)	1071 (44.4)	
1 to < 2 drinks/day, n (%)	1070 (11.1)	204 (8.5)	249 (10.3)	268 (11.1)	349 (14.5)	
2 to < 3 drinks/day, n (%)	447 (4.6)	74 (3.1)	72 (3.0)	105 (4.4)	196 (8.1)	
≥ 3 drinks/day, n (%)	896 (9.3)	88 (3.6)	173 (7.2)	235 (9.8)	400 (16.6)	
Smoking status						
Never smoker, n (%)	5640 (58.5)	1779 (73.8)	1586 (65.6)	1295 (53.8)	980 (40.6)	< 0.001
Past smoker, n (%)	1665 (17.3)	316 (13.1)	369 (15.3)	446 (18.5)	534 (22.1)	
Current smoker, n (%)	2340 (24.3)	317 (13.1)	461 (19.1)	664 (27.6)	898 (37.2)	
Educational level						
Elementary school or below, n (%)	1080 (11.2)	149 (6.2)	261 (10.8)	302 (12.6)	368 (15.3)	< 0.001
Middle school, n (%)	824 (8.5)	145 (6.0)	167 (6.9)	244 (10.1)	268 (11.1)	
High school, n (%)	4004 (41.5)	1071 (44.4)	1046 (43.3)	961 (40.0)	926 (38.4)	
College or above, n (%)	3737 (38.7)	1047 (43.4)	942 (39.0)	898 (37.3)	850 (35.2)	
Occupation						
Managers/professionals, n (%)	1478 (15.3)	429 (17.8)	359 (14.9)	339 (14.1)	351 (14.6)	< 0.001
Office/clerical support workers, n (%)	1100 (11.4)	293 (12.1)	276 (11.4)	256 (10.6)	275 (11.4)	
Service/sales workers, n (%)	1431 (14.8)	351 (14.6)	355 (14.7)	351 (14.6)	374 (15.5)	
Skilled agricultural/forestry/fishery workers, n (%)	500 (5.2)	109 (4.5)	99 (4.1)	149 (6.2)	143 (5.9)	
Craft plant/machine operators or assemblers, n (%)	1109 (11.5)	162 (6.7)	246 (10.2)	309 (12.8)	392 (16.3)	
Elementary occupations, n (%)	718 (7.4)	161 (6.7)	191 (7.9)	191 (7.9)	175 (7.3)	
Soldiers or the unemployed, n (%)	3309 (34.3)	907 (37.6)	890 (36.8)	810 (33.7)	702 (29.1)	
Physical activity						
Inactive, n (%)	3795 (39.3)	880 (36.5)	936 (38.7)	968 (40.2)	1011 (41.9)	< 0.001
Minimally active, n (%)	3413 (35.4)	902 (37.4)	911 (37.7)	812 (33.8)	788 (32.7)	
Active, n (%)	2437 (25.3)	630 (26.1)	569 (23.6)	625 (26.0)	613 (25.4)	
Menopausal status (for women)						
Premenopausal, n (%)	4623 (86.7)	1655 (94.0)	1332 (87.9)	973 (81.1)	663 (77.5)	< 0.001
Postmenopausal, n (%)	708 (13.3)	105 (6.0)	183 (12.1)	227 (18.9)	193 (22.5)	
Grain consumption						
< 2 times/day, n (%)	874 (9.1)	270 (11.2)	212 (8.8)	207 (8.6)	185 (7.7)	0.001
2 to < 3 times/day, n (%)	2759 (28.6)	724 (30.0)	698 (28.9)	658 (27.4)	679 (28.2)	
3 to < 4 times/day, n (%)	2630 (27.3)	606 (25.1)	675 (27.9)	666 (27.7)	683 (28.3)	
4 to < 5 times/day, n (%)	1322 (13.7)	349 (14.5)	322 (13.3)	341 (14.2)	310 (12.9)	
≥ 5 times/day, n (%)	2060 (21.4)	463 (19.2)	509 (21.1)	533 (22.2)	555 (23.0)	
Fish consumption						
< 1 time/week, n (%)	1230 (12.8)	309 (12.8)	306 (12.7)	305 (12.7)	310 (12.9)	0.829
1 to < 3 times/week, n (%)	2680 (27.8)	671 (27.8)	662 (27.4)	675 (28.1)	672 (27.9)	
3 to < 5 times/week, n (%)	1979 (20.5)	508 (21.1)	505 (20.9)	478 (19.9)	488 (20.2)	
5 times/week to < 1 time/day, n (%)	1168 (12.1)	270 (11.2)	289 (12.0)	286 (11.9)	323 (13.4)	
≥ 1 time/day, n (%)	2588 (26.8)	654 (27.1)	654 (27.1)	661 (27.5)	619 (25.7)	
Seaweed consumption						
< 1 time/week, n (%)	1577 (16.4)	390 (16.2)	384 (15.9)	408 (17.0)	395 (16.4)	0.696
1 to < 3 times/week, n (%)	2380 (24.7)	584 (24.2)	622 (25.7)	599 (24.9)	575 (23.8)	
3 to < 5 times/week, n (%)	2674 (27.7)	687 (28.5)	647 (26.8)	688 (28.6)	652 (27.0)	
5 times/week to < 1 time/day, n (%)	1271 (13.2)	313 (13.0)	315 (13.0)	300 (12.5)	343 (14.2)	
≥ 1 time/day, n (%)	1743 (18.1)	438 (18.2)	448 (18.5)	410 (17.0)	447 (18.5)	
Vegetable consumption						
< 1 time/day, n (%)	1216 (12.6)	363 (15.0)	328 (13.6)	286 (11.9)	239 (9.9)	< 0.001
1 to < 2 times/day, n (%)	1734 (18.0)	489 (20.3)	448 (18.5)	401 (16.7)	396 (16.4)	
2 to < 3 times/day, n (%)	3016 (31.3)	769 (31.9)	771 (31.9)	733 (30.5)	743 (30.8)	
3 to < 4 times/day, n (%)	3221 (33.4)	688 (28.5)	758 (31.4)	870 (36.2)	905 (37.5)	
≥ 4 times/day, n (%)	458 (4.7)	103 (4.3)	111 (4.6)	115 (4.8)	129 (5.3)	
Mushroom consumption						
Almost none, n (%)	1551 (16.1)	372 (15.4)	359 (14.9)	404 (16.8)	416 (17.2)	0.229
< 2 times/month, n (%)	1990 (20.6)	481 (19.9)	473 (19.6)	512 (21.3)	524 (21.7)	
2 times/month to < 1 time/week, n (%)	1855 (19.2)	468 (19.4)	490 (20.3)	454 (18.9)	443 (18.4)	
1 to < 3 times/week, n (%)	1959 (20.3)	486 (20.1)	516 (21.4)	486 (20.2)	471 (19.5)	
≥ 3 times/week, n (%)	2290 (23.7)	605 (25.1)	578 (23.9)	549 (22.8)	558 (23.1)	
Blood Pb concentration, mean ± SE, μg/dL	2.3 ± 0	2.0 ± 0	2.2 ± 0	2.4 ± 0	2.6 ± 0	< 0.001
Blood Hg concentration, mean ± SE, μg/L	4.5 ± 0.1	4.0 ± 0.1	4.1 ± 0.1	4.6 ± 0.1	5.3 ± 0.1	< 0.001
Blood Cd concentration, mean ± SE, μg/L	1.1 ± 0	1.0 ± 0	1.1 ± 0	1.2 ± 0	1.2 ± 0	< 0.001

TyG index, triglyceride glucose index; SE, standard error; BMI, body mass index; Pb, lead; Hg, mercury; Cd, cadmium.

^a^*p*-value was calculated using ANOVA tests for continuous variables and Rao-Scott Chi-Square tests for categorical variables.

### Distribution of blood concentrations of heavy metals

The GMs (95% CIs) of blood Pb, Hg, and Cd concentrations were 2.06 μg/dL (2.03–2.09), 3.69 μg/L (3.63–3.76), and 0.94 μg/L (0.93–0.96), respectively (Table 2). When divided by sex, men had higher blood concentrations of Pb and Hg but lower blood Cd concentrations than women.

**Table 2:** Distribution of blood Pb, Hg, and Cd concentrations according to sex.

	GM (95% CI)	Min	10%	25th	Median	75th	90%	Max
Pb, μg/dL								
Overall	2.06 (2.03, 2.09)	0.08	1.15	1.53	2.08	2.79	3.64	26.51
Men	2.43 (2.39, 2.47)	0.08	1.43	1.86	2.45	3.20	4.15	26.51
Women	1.78 (1.76, 1.81)	0.18	1.02	1.36	1.79	2.39	3.05	24.53
Hg, μg/L								
Overall	3.69 (3.63, 3.76)	0.22	1.73	2.46	3.63	5.51	8.06	168.49
Men	4.36 (4.26, 4.46)	0.32	1.98	2.91	4.34	6.55	9.29	168.49
Women	3.20 (3.14, 3.26)	0.22	1.58	2.20	3.18	4.54	6.64	38.50
Cd, μg/L								
Overall	0.94 (0.93, 0.96)	0.04	0.42	0.64	0.99	1.46	2.02	11.05
Men	0.88 (0.86, 0.90)	0.04	0.38	0.59	0.91	1.37	1.95	11.05
Women	1.00 (0.99, 1.02)	0.07	0.46	0.69	1.05	1.52	2.05	6.52

Pb, lead; Hg, mercury; Cd, cadmium; GM, geometric mean; CI, confidence interval; Min, minimum; Max, maximum.

### Association between blood heavy metals and TyG index

The associations between blood Pb concentrations and the TyG index according to the six cut-off points are shown in Table 3 and Supplementary Table S1. Briefly, a positive association was observed between blood Pb concentrations and the TyG index in both the continuous and categorical models using the six cut-off points. For example, as blood Pb concentrations increased, the prevalence of high TyG index increased by 23% and 25% the cut-off points 1 (95% CI = 1.06–1.44) and 2 (95% CI = 1.04–1.51), respectively. Likewise, participants in the highest quartile of blood Pb concentration had a higher TyG index than those in the lowest quartile (odds ratio [OR] [95% CI] 1.32 [1.07–1.63] and 1.46 [1.13–1.90] for cut-off points 1 and 2, respectively), with a significant linear trend. Notably, a stronger association between blood Pb concentrations and the TyG index was identified in men, whereas a null association was found in women. We observed consistent results when further adjusting for blood Hg and Cd concentrations.

**Table 3:** Associations between blood Pb, Hg, and Cd concentrations and the TyG index according to sex (for the cut-off point 1).

	Overall				Men				Women			
	Case/total	OR (95% CI)^a^	OR (95% CI)^b^	OR (95% CI)^c^	Case/total	OR (95% CI)^a^	OR (95% CI)^b^	OR (95% CI)^c^	Case/total	OR (95% CI)^a^	OR (95% CI)^b^	OR (95% CI)^c^
Pb												
Continuous	2412/9645	2.40 (2.11, 2.72)	1.23 (1.06, 1.44)	1.21 (1.03, 1.41)	1556/4314	1.97 (1.66, 2.34)	1.41 (1.14, 1.74)	1.32 (1.07, 1.64)	856/5331	1.63 (1.35, 1.97)	1.06 (0.85, 1.34)	1.12 (0.88, 1.41)
Quartile 1	372/2411	1.00 (Reference)	1.00 (Reference)	1.00 (Reference)	132/523	1.00 (Reference)	1.00 (Reference)	1.00 (Reference)	240/1888	1.00 (Reference)	1.00 (Reference)	1.00 (Reference)
Quartile 2	485/2410	1.43 (1.19, 1.71)	1.09 (0.89, 1.33)	1.08 (0.88, 1.32)	260/886	1.46 (1.09, 1.94)	1.28 (0.93, 1.76)	1.24 (0.90, 1.71)	225/1524	1.18 (0.93, 1.50)	0.99 (0.77, 1.29)	1.03 (0.79, 1.34)
Quartile 3	671/2412	2.05 (1.73, 2.43)	1.19 (0.97, 1.46)	1.17 (0.95, 1.43)	451/1245	1.92 (1.48, 2.50)	1.46 (1.07, 2.00)	1.38 (1.01, 1.89)	220/1167	1.53 (1.19, 1.96)	1.00 (0.75, 1.33)	1.04 (0.78, 1.39)
Quartile 4	884/2412	3.01 (2.55, 3.55)	1.32 (1.07, 1.63)	1.28 (1.03, 1.59)	713/1660	2.55 (1.97, 3.30)	1.71 (1.24, 2.35)	1.57 (1.13, 2.17)	171/752	1.73 (1.33, 2.24)	1.05 (0.76, 1.44)	1.11 (0.81, 1.53)
*p* for trend		< 0.001	0.006	0.015		< 0.001	0.001	0.004		< 0.001	0.817	0.549
Hg												
Continuous	2412/9645	1.64 (1.50, 1.80)	1.01 (0.91, 1.12)	0.99 (0.89, 1.10)	1556/4314	1.64 (1.45, 1.86)	1.13 (0.98, 1.30)	1.09 (0.95, 1.25)	856/5331	1.04 (0.89, 1.21)	0.83 (0.70, 0.98)	0.83 (0.70, 0.98)
Quartile 1	433/2411	1.00 (Reference)	1.00 (Reference)	1.00 (Reference)	177/681	1.00 (Reference)	1.00 (Reference)	1.00 (Reference)	256/1730	1.00 (Reference)	1.00 (Reference)	1.00 (Reference)
Quartile 2	521/2411	1.30 (1.10, 1.53)	1.06 (0.87, 1.27)	1.04 (0.86, 1.25)	276/902	1.42 (1.10, 1.84)	1.26 (0.94, 1.67)	1.21 (0.91, 1.62)	245/1509	1.09 (0.87, 1.36)	0.99 (0.78, 1.26)	0.99 (0.78, 1.26)
Quartile 3	643/2412	1.62 (1.37, 1.92)	1.04 (0.86, 1.27)	1.01 (0.83, 1.23)	445/1185	1.93 (1.50, 2.47)	1.36 (1.02, 1.81)	1.29 (0.96, 1.71)	198/1227	1.03 (0.80, 1.31)	0.84 (0.64, 1.10)	0.83 (0.63, 1.10)
Quartile 4	815/2411	2.15 (1.84, 2.51)	1.01 (0.84, 1.22)	0.97 (0.81, 1.17)	658/1546	2.29 (1.81, 2.89)	1.30 (0.98, 1.71)	1.21 (0.92, 1.60)	157/865	1.03 (0.80, 1.33)	0.75 (0.56, 1.00)	0.75 (0.56, 1.00)
*p* for trend		< 0.001	0.992	0.670		< 0.001	0.127	0.318		0.866	0.033	0.036
Cd												
Continuous	2412/9645	1.43 (1.30, 1.57)	1.14 (1.01, 1.30)	1.12 (0.98, 1.27)	1556/4314	1.58 (1.40, 1.78)	1.36 (1.14, 1.61)	1.31 (1.10, 1.55)	856/5331	1.62 (1.37, 1.92)	0.91 (0.74, 1.13)	0.92 (0.75, 1.14)
Quartile 1	461/2412	1.00 (Reference)	1.00 (Reference)	1.00 (Reference)	332/1232	1.00 (Reference)	1.00 (Reference)	1.00 (Reference)	129/1180	1.00 (Reference)	1.00 (Reference)	1.00 (Reference)
Quartile 2	568/2412	1.31 (1.11, 1.54)	1.05 (0.86, 1.27)	1.03 (0.85, 1.25)	383/1104	1.44 (1.18, 1.77)	1.14 (0.90, 1.45)	1.10 (0.87, 1.40)	185/1308	1.36 (1.02, 1.81)	0.94 (0.68, 1.29)	0.95 (0.69, 1.30)
Quartile 3	649/2408	1.53 (1.31, 1.79)	1.08 (0.89, 1.32)	1.06 (0.87, 1.29)	415/1017	1.80 (1.46, 2.22)	1.28 (0.99, 1.65)	1.22 (0.94, 1.58)	234/1391	1.68 (1.28, 2.19)	0.83 (0.60, 1.15)	0.83 (0.60, 1.15)
Quartile 4	734/2413	1.82 (1.56, 2.13)	1.29 (1.04, 1.59)	1.24 (1.01, 1.53)	426/961	2.14 (1.73, 2.63)	1.74 (1.31, 2.31)	1.63 (1.22, 2.17)	308/1452	2.22 (1.71, 2.88)	0.88 (0.63, 1.24)	0.90 (0.64, 1.26)
*p* for trend		< 0.001	0.018	0.042		< 0.001	< 0.001	0.001		< 0.001	0.465	0.507

Pb, lead; Hg, mercury; Cd, cadmium; TyG index, triglyceride glucose index; OR, odds ratio; CI, confidence interval.

The cut-off point 1: 8.84 for the highest quartile of the TyG index in this study.

^a^Model 1, with no adjustment.

^b^Model 2, adjusted for age, sex (for men and women combined), survey year, BMI, alcohol consumption, smoking status, educational level, occupation, physical activity, menopausal status, grain consumption, fish consumption, seaweed consumption, vegetable consumption, and mushroom consumption.

^c^Model 3, further adjusted for natural log–transformed blood concentrations of Pb, Hg, or Cd.

A null association was identified between blood Hg concentrations and the TyG index using cut-off points 1 and 2 (Table 3). However, we found the association to be inconsistent between the lowest and the highest cut-off points. The prevalence of high TyG index tended to decrease in women when the lowest cut-off point 3 was applied, whereas it tended to increase in men when applying the highest cut-off point 6 (Supplementary Table S2).

As shown in Table 3 and Supplementary Table S3, the TyG index was positively associated with blood Cd concentrations. For instance, when we applied cut-off point 1, the OR (95% CI) for the prevalence of high TyG index was 1.14 (1.01–1.30) for natural log–transformed blood Cd concentrations. Similarly, compared with participants in the lowest quartile of blood Cd concentrations, those in the highest quartile had a 29% higher prevalence of high TyG index (95% CI = 1.04–1.59). When separated by sex, the positive association persisted in men but not in women.

In addition, Supplementary Tables S4 and S5 present results of subgroup analysis for 3678 participants who with HOMA-IR values. There was no significant association between blood heavy metals and HOMA-IR (Supplementary Table S4). While blood Pb and Cd concentrations were significantly associated with TyG index (OR [95% CI]: for overall, 1.43 [1.08–1.89] for natural log–transformed blood Pb concentrations; for men, 1.50 [1.02–2.20] and 1.40 [1.04–1.89] for natural log–transformed blood concentrations of Pb and Cd, respectively) (Supplementary Table S5).

## Discussion

We used a nationally representative data in Korea to identify the association of blood Pb, Hg, and Cd concentrations with the TyG index, a surrogate marker for IR and related cardiovascular-metabolic diseases. In summary, regardless of the cut-off points, the TyG index tended to increase with the increment of Pb and Cd blood concentrations. These positive associations were prominent in men. We also observed that the TyG index did not differ with blood Hg concentrations. The findings of this study indicate that management on priority heavy metals are imperative for prevention of IR and related cardiovascular-metabolic diseases.

Some previous studies have reported that concentrations of Pb and Cd measured in blood^16,17^, urine^16,18,19^, and adipose tissue^20^ were not significantly associated with IR (HOMA-IR). Especially, a Korean study that used data from the 2009–2010 Korean National Health and Nutrition Examination Survey (KNHANES) demonstrated that the HOMA-IR was not associated with blood concentrations of Pb, Hg, and Cd^17^. Incidentally, the composition of the previous study population differed significantly from that of the present study (Supplementary Table S6). While Moon SS's study comprised adult participants aged 30 years and older, which might influence the HOMA-IR value, our study encompassed adult participants aged 19 years and older, excluding those with T2DM, hyperlipidemia, hypertension, stroke, myocardial infarction, and cancer. Consequently, the GMs of HOMA-IR were higher than that in our study (2.25 vs. 2.06 for Moon SS’s study vs. our study), which resulted in a higher prevalence of HOMA-IR (36.5% vs. 28.5% for Moon SS’ study vs. our study). Similarly, the prevalence of elevated TyG index in dataset of Moon SS’s study was also higher than our study when applying all the 4 cut-off points referred in this study. The misclassification of outcomes in the Moon SS’s study may underestimate the effect size of associations.

On the other hand, the present study, which included 3678 participants with HOMA-IR values from KNHANES 2008–2010, indicated no significant association between blood heavy metals and HOMA-IR, consistent with Moon SS's study. While significant associations of blood Pb and Cd with TyG index were observed among 3678 participants with HOMA-IR. In light of these findings, the significant associations between blood Pb and Cd concentrations and the TyG index in this study might to be due to the better performance of the TyG index than the HOMA-IR and other indices when predicting IR. According to a study on validation of TyG index, it had higher sensitivity and specificity than the HOMA-IR in identifying IR patients (area under the receiver operating characteristic curve: 0.79 for the TyG index and 0.77 for the HOMA-IR)^10^. Furthermore, in the TyG index equation, the fat distribution is corrected so that the patients with normal weight but displaying obesity-related metabolic derangements can also be applied. However, the HOMA-IR model has poor detect power in cases other than insulin signaling pathway defects such as impaired hepatocyte function^21^.

On the other hand, concentrations of Pb and Cd have been reported to be associated with IR-related cardiovascular-metabolic disorders. Positive associations have also been observed between Pb and Cd exposures and prevalence^5^, incidence^22^, and mortality^23^ of CVDs. A meta-analysis of 37 case–control and prospective studies concluded that the risk of CVDs increased by 43% and 33% with the increment of Pb and Cd levels, respectively^4^. Exposure to Pb and Cd may also be related to MetS^24^, especially in the Asian population^25^. Furthermore, several observational and meta-analyses have demonstrated the positive association between Pb exposure and dyslipidemia^26,27^ and between Cd exposure and T2DM^19,28^. However, the literature shows inconsistent results on the association between environmental Hg exposure and IR^17,29,30^. A study in Taiwan reported that blood Hg concentrations were associated with elevated HOMA-IR^29^, whereas the positive association was observed only in men in a Korean population^30^. A systematic review reported that the inconsistent results of previous longitudinal studies make it difficult to infer causality between Hg exposure and T2DM or MetS^31^. Further evaluations are needed to explore the effects of heavy metal exposure on the risk of IR and related cardiovascular-metabolic disorders in large prospective studies.

Several studies have suggested pathological mechanisms underlying the toxicity of heavy metals on IR progression. The heavy metals discussed in this study are considered hyperglycemic metals^32^. When these heavy metals accumulate in the body, they promote direct damage to pancreatic β-cells by interacting with the plasma calcium channel and altering intracellular calcium homeostasis^33^. Accordingly, the inhibited calcium-ATPase may further affect insulin biosynthesis and secretion^34^. As another potential mechanism of oxidative stress, heavy metals in the target tissues may stimulate the production of reactive oxygen species and depletion of the antioxidant pool^35^. Heavy metal–induced oxidative stress results in the reduction of insulin sensitivity and pancreatic β-cell function, which is further linked to the progression of T2DM^21^. It has also been suggested that chronic and sub-chronic exposure to Cd could reduce mRNA and protein expression of the insulin-regulated glucose transporter type 4 (GLUT4) in muscle and adipose tissues^36,37^. Consequently, most glucose uptake occurs in other insulin-independent tissues, such as the liver, kidney, and brain^38^. IR may be developed when insulin secretion from pancreatic β-cells increases to compensate for the hyperglycemic state^39^. Moreover, several in vivo and in vitro studies have reported genotoxicity of the heavy metals via stimulation of DNA breakage and modulation of mRNA expression^40^. Nevertheless, detailed evidence of the effect of heavy metal exposures on IR and its complications is limited, warranting further experimental and epidemiological investigations.

The Human Biomonitoring (HBM) Commission of the German Federal Environmental Agency has recently defined the reference values for heavy metal concentrations in blood: 9.00 µg/dL in men and 7.00 µg/dL in women for Pb; 2.00 µg/L for Hg; and 1.00 µg/L for Cd^41^ (Supplementary Figure S1). The GMs of blood Pb and Cd concentrations among this study’s participants (2.06 µg/dL and 0.94 µg/L, respectively) were lower than the HBM Commission’s reference values. Likewise, the concentrations of Pb and Cd in other countries have also been found to fall below the reference values: 1.32 µg/dL Pb and 0.37 µg/L Cd (GMs in blood) in the US^42^; 1.88 µg/dL Pb and 0.39 µg/L Cd (GMs in blood) in Northern France^43^; 2.40 µg/dL Pb (GM in blood) and 0.28 µg/L Cd (GM in urine) in Spain^44,45^; and 2.33 µg/dL Pb and 0.82 µg/L Cd (medians in blood) in China^46^. Given the significant positive association of blood Pb and Cd concentrations with the TyG index in this study, IR and related cardiovascular-metabolic disorders may be induced even at low-dose exposures of Pb and Cd^47^. By contrast, the GMs of blood Hg concentrations in this study participants (3.69 µg/L) and Spanish adults (6.35 µg/L)^48^ were higher than the HBM Commission’s reference value, whereas concentrations below the reference value were assessed among individuals in the US (0.93 µg/L [GM in blood])^42^, Northern France (1.38 µg/L [GM in blood])^43^, and China (1.17 µg/L [median in blood])^46^. The null association between blood Hg concentrations and the TyG index in this study could be interpreted as a high distribution of Hg exposure, as a non-linear dose–response relationship between Hg concentrations and risk of hypertension was observed in previous studies^49^.

With this study, we are the first to evaluate the association between Pb, Hg, and Cd exposure and TyG index. The large sample size from nationally representative survey data supports the external validity of the results in this study. Additionally, the cut-off points of the TyG index determined in different populations were applied to minimize the probability of misclassification. However, several limitations remain. First, the findings of this cross-sectional study may not offer causal insights into the effect of heavy metal exposure on the risk of IR and related complications. Second, we did not evaluate the additive effect of Pb, Hg, and Cd^24^. However, the association between blood concentrations of heavy metals and the TyG index persisted after adjusting for each. Finally, even though we controlled for all confounding factors considered, residual bias cannot be ruled out.

In conclusion, this study’s findings indicate that low dose environmental exposure to Pb and Cd—but not Hg—were associated with TyG index, a reliable surrogate marker for IR-related cardiovascular-metabolic diseases. Implementing of management policies for priority heavy metals to prevent IR and related cardiovascular-metabolic disorders is of great significance for public health.

## Methods

### Study population

The data used in this study were acquired from the KNHANES, a nationally representative health survey conducted by the Korea Disease Control and Prevention Agency (KDCA)^50^. Approximately 10000 individuals aged 1 year and older were selected annually via multi-stage clustered probability sampling. After a face-to-face interview and health examination, information on socioeconomic status, behavioral and nutritional factors, anthropometric indices, clinical profiles, and medical history was obtained from each participant.

**Figure 1:**
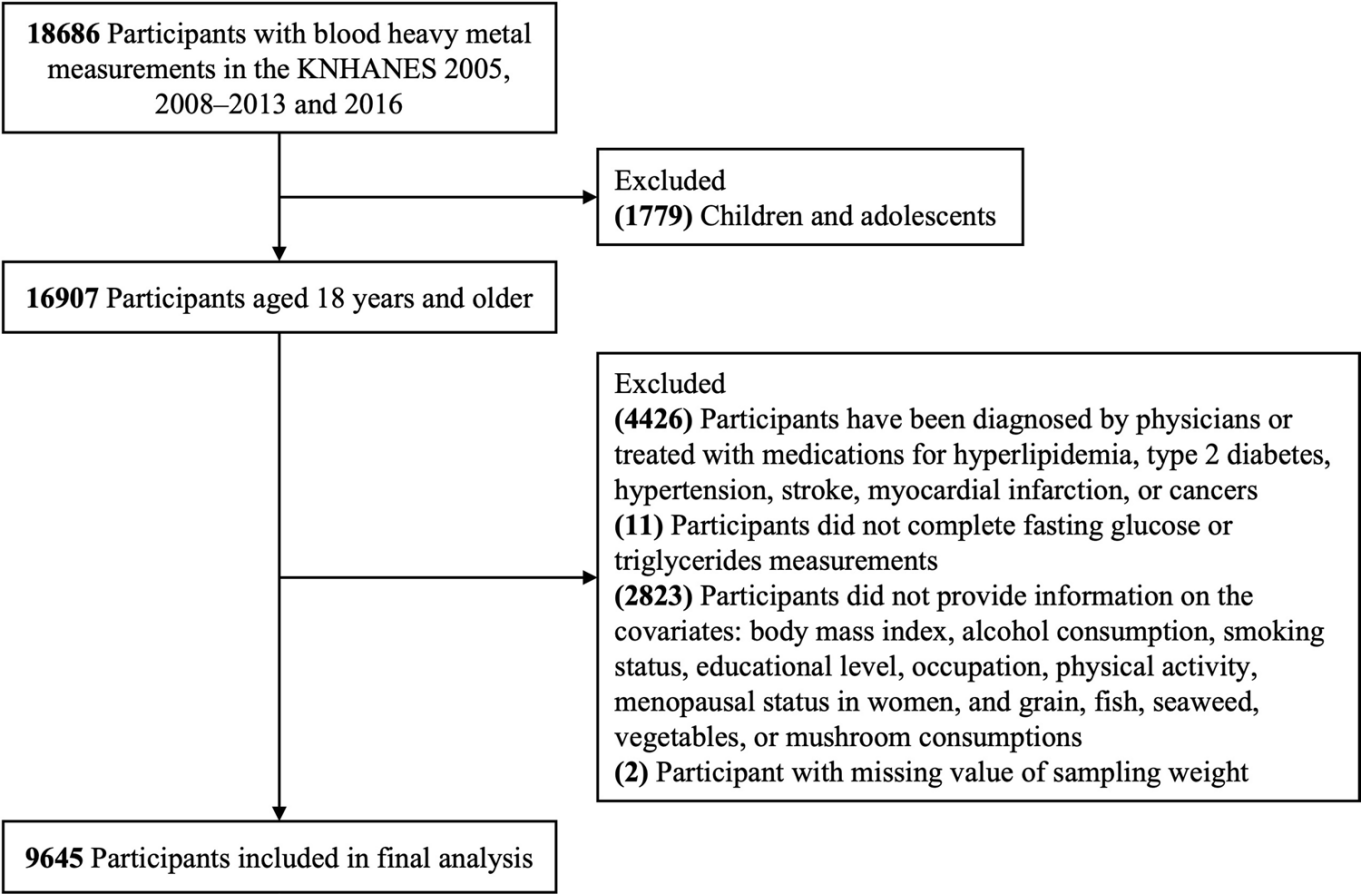
Flow diagram of the study participants.

In the KNHANES, blood heavy metal measurement was conducted in a randomly selected sample in 2005, 2008–2013, and 2016–2017. Participants except those who enrolled in 2017 were initially included due to the lack of information on food frequency questionnaire (FFQ). Of the 18686 participants, 1779 children and adolescents were excluded. We further excluded participants who had received a physician’s diagnosis of or been treated with medication for hyperlipidemia (n = 1697), T2DM (n = 726), hypertension (n = 1673), stroke (n = 60), myocardial infarction (n = 20), or cancer (n = 250); those who did not complete fasting plasma glucose (FPG) (n = 10) or TG (n = 1) measurements; those who did not provide information on body mass index (BMI) (n = 36), alcohol consumption (n = 320), smoking status (n = 5), educational level (n = 94), occupation (n = 8), physical activity (n = 4), menopausal status in women (n = 228), or grain, fish, seaweed, vegetable, or mushroom consumption (n = 2128); and those with a missing sampling weight (n = 2). After exclusion, 9645 participants (4314 men and 5331 women) were included in the final analysis. A flow diagram of the inclusion and exclusion criteria for the study population is shown in Fig. 1.

This study was performed in accordance with the principles of the Declaration of Helsinki. The protocol was reviewed and approved by the Institutional Review Board of the KDCA (2008-04EXP-01-C, 2009-01CON-03-2C, 2010-02CON-21-C, 2011-02CON-06-C, 2012-01EXP-01-2C, 2013-07CON-03-4C, 2015-01-02-6C). Informed consent was submitted by all participants when they were enrolled.

### Clinical assessment

Participants were instructed to fast for at least 8 h before the blood was drawn. Exposure to Pb (µg/dL) and Cd (µg/L) in whole blood samples was assessed by graphite furnace atomic absorption spectrometry methods using the AAnalyst 600 (PerkinElmer, Turku, Finland). Blood Hg concentrations (µg/L) were measured via the gold-amalgam collection method with a DMA-80 (Milestone, Bergamo, Italy). The limits of detection (LOD) for blood Pb, Hg, and Cd for each round of survey were shown in Supplementary Table S7^51–55^. A total of 28 participants with blood Pb, Hg, or Cd concentrations below the LOD were assigned a value of the LOD divided by the square root of 2. FPG (mg/dL) and TG (mg/dL) levels were determined by the hexokinase UV method and enzymatic method using the Hitachi Automatic Analyzer 7600-210 (Hitachi, Tokyo, Japan). Information on the KNHANES quality control program can be found in prior publications^55^.

### Case ascertainment

The TyG index was calculated using the following equation: TyG index = Ln (FPG [mg/dL] × TG [mg/dL]/2). In this study, we adopted six cut-off points to define the high TyG index group. First, the highest quartile of the TyG index in this study (abbreviated as cut-off point 1) was defined by a cut-off point of 8.84. We also applied four TyG index cut-off points proposed in previous studies: (1) 9.03 for incident CVDs in an Iranian population (cut-off point 2)^14^; (2) 8.49 in men and 8.12 in women for IR in a Japanese population (cut-off point 3)^11^; (3) 8.76 for IR in a Chinese population (cut-off point 4)^12^; and (4) 8.52 for MetS in a Korean population (cut-off point 5)^13^. Furthermore, we included a calculated TyG index cut-off point of 9.44 (cut-off point 6) according to the American Diabetes Association and the Third Report of the Expert Panel on Detection, Evaluation, and Treatment of High Blood Cholesterol in Adults by the National Cholesterol Education Program criteria for T2DM (FPG ≥ 126 mg/dL) and hypertriglyceridemia (TG ≥ 200 mg/dL)^56,57^.

For subgroup analysis among 3678 participants with HOMA-IR values, the HOMA-IR was calculated using the following equation: HOMA-IR = FPG (mmol/l) × fasting insulin (mIU/l) ÷ 22.5. Participants with HOMA-IR ≥ 2.5 were defined as insulin resistance^58^. A cut-off point of 8.828 for TyG index (the highest quartile of the among these participants) was applied.

### Covariates

BMI (kg/m^2^) was calculated by dividing each participant’s body weight (kg) by the square of their height (m). Per the obesity guidelines in the Asia–Pacific region by the World Health Organization Western Pacific Office, participants were classified as normal weight (BMI < 23.0 kg/m^2^), overweight (BMI 23.0–24.9 kg/m^2^), or obese (BMI ≥ 25.0 kg/m^2^). Alcohol consumption was calculated in drinks/day regardless of the type of alcoholic beverage. Participants’ current occupations were categorized according to the 6^th^ version of the Korean Standard Classification of Occupation. Physical activity was evaluated using the International Physical Activity Questionnaire and the Global Physical Activity Questionnaire. Participants were categorized as inactive, minimally active, or active based on their metabolic equivalent task (MET)-min per week. Dietary grain, fish, seaweed, vegetable, and mushroom consumption in times/day were assessed using a validated FFQ.

### Statistical analysis

Due to the skewed distribution, the blood concentrations of Pb, Hg, and Cd were converted to natural log–transformed values. Baseline descriptions of the study participants were presented as mean (standard error) or frequency (percentage). Differences in baseline characteristics according to quartiles of the TyG index were determined using ANOVA tests for continuous variables and Rao-Scott Chi-Square tests for categorical variables. We calculated the GMs and 95% CIs of the heavy metal concentrations using PROC SURVEYMEANS.

ORs and 95% CIs for the associations between blood Pb, Hg, and Cd concentrations and the TyG index with the six cut-off points were estimated using PROC SURVEYLOGISTIC. The natural log–transformed blood Pb, Hg, and Cd concentrations were used in the association analyses. Categorical models using the quartiles of blood concentrations of Pb, Hg, and Cd were also applied, with the lowest quartile used as the reference group. The median value of the blood heavy metal concentrations was assigned to each quartile group to evaluate the linear trend.

To control the potential confounding factors, we examined several possible covariates and selected the variables whose ORs changed by more than 7–10% after adjustment. Moreover, we also added the food groups containing in heavy metals as confounding factors. As a result, three models were estimated in this study: model 1, with no adjustment; model 2, adjusted for age (years, continuous), sex (for men and women combined), survey year (2005, 2008, 2009, 2010, 2011, 2012, 2013, and 2016), BMI (kg/m^2^, continuous), alcohol consumption (non-drinkers, < 1 drink/day, 1 to < 2 drinks/day, 2 to < 3 drinks/day, and ≥ 3 drinks/day), smoking status (never smoker, past smoker, and current smoker), educational level (elementary school or below, middle school, high school, and college or above), occupation (managers/professionals, office/clerical support workers, service/sales workers, skilled agricultural/forestry/fishery workers, craft plant/machine operators or assemblers, elementary occupations, and soldiers or the unemployed), physical activity (inactive, minimally active, and active), menopausal status (for women, premenopausal and postmenopausal), grain consumption (< 2 times/day, 2 to < 3 times/day, 3 to < 4 times/day, 4 to < 5 times/day, and ≥ 5 times/day), fish consumption (< 1 time/week, 1 to < 3 times/week, 3 to < 5 times/week, 5 times/week to < 1 time/day, and ≥ 1 time/day), seaweed consumption (< 1 time/week, 1 to < 3 times/week, 3 to < 5 times/week, 5 times/week to < 1 time/day, and ≥ 1 time/day), vegetable consumption (< 1 time/day, 1 to < 2 times/day, 2 to < 3 times/day, 3 to < 4 times/day, and ≥ 4 times/day), and mushroom consumption (almost none, < 2 times/month, 2 times/month to < 1 time/week, 1 to < 3 times/week, and ≥ 3 times/week); and model 3, adjusted additionally for natural log–transformed blood concentrations of Pb, Hg, or Cd. For instance, in model 3, natural log–transformed blood Hg and Cd concentrations were adjusted for the association between blood Pb concentration and the TyG index.

All analyses in this study were conducted with a sampling weight. Statistical analyses were conducted using SAS version 9.4 (SAS Institute Inc., Cary, NC, USA). *P*-values < 0.05 were considered to indicate significant differences.

### Supplementary Information

Supplementary Information.

## Data Availability

The datasets generated and analyzed during this study are available in the KNHANES repository, https://knhanes.kdca.go.kr/knhanes/eng/index.do.

## References

[1] Jaishankar M, Tseten T, Anbalagan N, Mathew BB, Beeregowda KN (2014). Toxicity, mechanism and health effects of some heavy metals. Interdiscip. Toxicol..

[2] He ZL, Yang XE, Stoffella PJ (2005). Trace elements in agroecosystems and impacts on the environment. J. Trace Elem. Med. Biol..

[3] United States Environmental Protection Agency. *Priority Pollutant List*, <https://www.epa.gov/sites/default/files/2015-09/documents/priority-pollutant-list-epa.pdf> (2014).

[4] Chowdhury R (2018). Environmental toxic metal contaminants and risk of cardiovascular disease: Systematic review and meta-analysis. BMJ.

[5] Agarwal S, Zaman T, Murat Tuzcu E, Kapadia SR (2011). Heavy metals and cardiovascular disease: Results from the national health and nutrition examination survey (NHANES) 1999–2006. Angiology.

[6] Hendryx M, Luo J, Chojenta C, Byles JE (2021). Exposure to heavy metals from point pollution sources and risk of incident type 2 diabetes among women: A prospective cohort analysis. Int. J. Environ. Health Res..

[7] Tsoi MF, Lo CWH, Cheung TT, Cheung BMY (2021). Blood lead level and risk of hypertension in the United States National Health and Nutrition Examination Survey 1999–2016. Sci. Rep..

[8] Wu W (2018). Associations of environmental exposure to metals with the risk of hypertension in China. Sci. Total Environ..

[9] Moon MK (2022). Lead, mercury, and cadmium exposures are associated with obesity but not with diabetes mellitus: Korean National Environmental Health Survey (KoNEHS) 2015–2017. Environ. Res..

[10] Vasques ACJ (2011). TyG index performs better than HOMA in a Brazilian population: A hyperglycemic clamp validated study. Diabetes Res. Clin. Pract..

[11] Nakagomi A, Sunami Y, Kawasaki Y, Fujisawa T, Kobayashi Y (2020). Sex difference in the association between surrogate markers of insulin resistance and arterial stiffness. J. Diabetes Complicat..

[12] Huang R (2022). Usefulness of four surrogate indexes of insulin resistance in middle-aged population in Hefei, China. Ann. Med..

[13] Son D-H, Lee HS, Lee Y-J, Lee J-H, Han J-H (2022). Comparison of triglyceride-glucose index and HOMA-IR for predicting prevalence and incidence of metabolic syndrome. Nutrit. Metab. Cardiovasc. Dis..

[14] Barzegar N, Tohidi M, Hasheminia M, Azizi F, Hadaegh F (2020). The impact of triglyceride-glucose index on incident cardiovascular events during 16 years of follow-up: Tehran Lipid and Glucose Study. Cardiovasc. Diabetol..

[15] Tahapary DL (2022). Challenges in the diagnosis of insulin resistance: Focusing on the role of HOMA-IR and Tryglyceride/glucose index. Diabetes Metab. Syndr. Clin. Res. Rev..

[16] Barregard L, Bergström G, Fagerberg B (2013). Cadmium exposure in relation to insulin production, insulin sensitivity and type 2 diabetes: A cross-sectional and prospective study in women. Environ. Res..

[17] Moon SS (2013). Association of lead, mercury and cadmium with diabetes in the Korean population: The Korea National Health and Nutrition Examination Survey (KNHANES) 2009–2010. Diabetic Med..

[18] Menke A, Guallar E, Cowie CC (2015). Metals in urine and diabetes in U.S. Adults. Diabetes.

[19] Wallia A, Allen NB, Badon S, El Muayed M (2014). Association between urinary cadmium levels and prediabetes in the NHANES 2005–2010 population. Int. J. Hyg. Environ. Health.

[20] Salcedo-Bellido I (2021). Adipose tissue cadmium concentrations as a potential risk factor for insulin resistance and future type 2 diabetes mellitus in GraMo adult cohort. Sci. Total Environ..

[21] Khan AR, Awan FR (2014). Metals in the pathogenesis of type 2 diabetes. J. Diabetes Metab. Disord..

[22] Choi S, Kwon J, Kwon P, Lee C, Jang S-I (2020). Association between blood heavy metal levels and predicted 10-year risk for a first atherosclerosis cardiovascular disease in the general Korean population. Int. J. Environ. Res. Public Health.

[23] Larsson SC, Wolk A (2016). Urinary cadmium and mortality from all causes, cancer and cardiovascular disease in the general population: Systematic review and meta-analysis of cohort studies. Int. J. Epidemiol..

[24] Moon S-S (2014). Additive effect of heavy metals on metabolic syndrome in the Korean population: The Korea National Health and Nutrition Examination Survey (KNHANES) 2009–2010. Endocrine.

[25] Lu L (2022). Associations of cadmium exposure with risk of metabolic syndrome and its individual components: A meta-analysis. J. Expos. Sci. Environ. Epidemiol..

[26] Xu H, Mao Y, Xu B, Hu Y (2021). Low-level environmental lead and cadmium exposures and dyslipidemia in adults: Findings from the NHANES 2005–2016. J. Trace Elem. Med. Biol..

[27] Zhu X (2021). Association between blood heavy metal concentrations and dyslipidemia in the elderly. Biol. Trace Elem. Res..

[28] Schwartz GG, Il’yasova D, Ivanova A (2003). Urinary cadmium, impaired fasting glucose, and diabetes in the NHANES III. Diabetes Care.

[29] Chang J-W (2011). Simultaneous exposure of non-diabetics to high levels of dioxins and mercury increases their risk of insulin resistance. J. Hazard. Mater..

[30] Kim K-N, Park S-J, Choi B, Joo N-S (2015). Blood mercury and insulin resistance in nondiabetic Koreans (KNHANES 2008–2010). ymj.

[31] Roy C, Tremblay P-Y, Ayotte P (2017). Is mercury exposure causing diabetes, metabolic syndrome and insulin resistance? A systematic review of the literature. Environ. Res..

[32] González-Villalva A (2016). Pollution by metals: Is there a relationship in glycemic control?. Environ. Toxicol. Pharmacol..

[33] Levy J (1999). Abnormal cell calcium homeostasis in type 2 diabetes mellitus. Endocrine.

[34] Hectors TLM (2011). Environmental pollutants and type 2 diabetes: A review of mechanisms that can disrupt beta cell function. Diabetologia.

[35] Ercal N, Gurer-Orhan H, Aykin-Burns N (2001). Toxic metals and oxidative stress part I: Mechanisms involved in metal-induced oxidative damage. Curr. Top. Med. Chem..

[36] Bryant NJ, Govers R, James DE (2002). Regulated transport of the glucose transporter GLUT4. Nat. Rev. Mol. Cell Biol..

[37] Han JC (2003). Cadmium induces impaired glucose tolerance in rat by down-regulating GLUT4 expression in adipocytes. Arch. Biochem. Biophys..

[38] DeFronzo RA (2004). Pathogenesis of type 2 diabetes mellitus. Medical Clinics.

[39] Treviño S (2015). Chronic cadmium exposure in rats produces pancreatic impairment and insulin resistance in multiple peripheral tissues. Arch. Biochem. Biophys..

[40] Eke D, Çelik A (2008). Genotoxicity of thimerosal in cultured human lymphocytes with and without metabolic activation sister chromatid exchange analysis proliferation index and mitotic index. Toxicol. Vitro.

[41] Apel P, Angerer J, Wilhelm M, Kolossa-Gehring M (2017). New HBM values for emerging substances, inventory of reference and HBM values in force, and working principles of the German Human Biomonitoring Commission. Int. J. Hygiene Environ. Health.

[42] Wang X, Mukherjee B, Park SK (2018). Associations of cumulative exposure to heavy metal mixtures with obesity and its comorbidities among US adults in NHANES 2003–2014. Environ. Int..

[43] Nisse C (2017). Blood and urinary levels of metals and metalloids in the general adult population of Northern France: The IMEPOGE study, 2008–2010. Int. J. Hygiene Environ. Health.

[44] Cañas AI (2014). Blood lead levels in a representative sample of the Spanish adult population: The BIOAMBIENT.ES project. Int. J. Hygiene Environ. Health.

[45] López-Herranz A (2016). Cadmium levels in a representative sample of the Spanish adult population: The BIOAMBIENT.ES project. J. Expos. Sci. Environ. Epidemiol..

[46] Qu Y (2022). Effect of exposures to mixtures of lead and various metals on hypertension, pre-hypertension, and blood pressure: A cross-sectional study from the China National Human Biomonitoring. Environ. Pollut..

[47] Zhou J, Meng X, Deng L, Liu N (2022). Non-linear associations between metabolic syndrome and four typical heavy metals: Data from NHANES 2011–2018. Chemosphere.

[48] Castaño A (2019). Mercury levels in blood, urine and hair in a nation-wide sample of Spanish adults. Sci. Total Environ..

[49] Park SK, Lee S, Basu N, Franzblau A (2013). Associations of blood and urinary mercury with hypertension in US adults: The NHANES 2003–2006. Environ. Res..

[50] Kweon S (2014). Data resource profile: The Korea National Health and Nutrition Examination Survey (KNHANES). Int. J. Epidemiol..

[51] Korea Centers for Disease Control and Prevention. Clinical Laboratory Test for the Fourth Korea National Health and Nutrition Examination Survey (2007–2009). (Chengju, Korea, 2009).

[52] Korea Centers for Disease Control and Prevention. Clinical Laboratory Test for the Fifth Korea National Health and Nutrition Examination Survey (2010–2012). (Chengju, Korea, 2012).

[53] Korea Centers for Disease Control and Prevention. Clinical Laboratory Test for the Sixth Korea National Health and Nutrition Examination Survey (2013–2015). (Chengju, Korea, 2015).

[54] Korea Centers for Disease Control and Prevention. Clinical Laboratory Test for the Seventh Korea National Health and Nutrition Examination Survey (2016–2018). (Chengju, Korea, 2018).

[55] Kim N-S, Lee B-K (2011). National estimates of blood lead, cadmium, and mercury levels in the Korean general adult population. Int. Arch. Occup. Environ. Health.

[56] American Diabetes Association (2010). Diagnosis and classification of diabetes mellitus. Diabetes Care.

[57] Expert Panel on Detection Evaluation and Treatment of High Blood Cholesterol in Adults (2001). Executive summary of the third report of the national cholesterol education program (NCEP) Expert panel on detection, evaluation, and treatment of high blood cholesterol in adults (adult treatment panel III). JAMA.

[58] Vardeny O (2013). Insulin resistance and incident heart failure: The ARIC study (atherosclerosis risk in communities). JACC Heart Fail..

