# Supplementary material for: Environmental exposure to lead and cadmium are associated with triglyceride glucose index
Source: Sci Rep. 2024 Jan 30;14:2496. doi: 10.1038/s41598-024-52994-5 (PMC10827717; doi:10.1038/s41598-024-52994-5)
Supplement: Supplementary file 1 — Supplementary Information. [file 41598_2024_52994_MOESM1_ESM.docx]

**Environmental exposure to lead and cadmium are associated with triglyceride glucose index**

Taiyue Jin, Eun Young Park, Byungmi Kim, and Jin-Kyoung Oh

**Supplementary Figure S1.** The reference values of blood Pb, Hg, and Cd concentrations recommended by the HBM Commission and the concentrations in Korea, USA, Northern France, Spain, and China.

**Supplementary Table S1.** Associations between blood Pb concentrations and the TyG index according to sex (for the cut-off points 2 to 6).

**Supplementary Table S2.** Associations between blood Hg concentrations and the TyG index according to sex (for the cut-off points 2 to 6).

**Supplementary Table S3.** Associations between blood Cd concentrations and the TyG index according to sex (for the cut-off points 2 to 6).

**Supplementary Table S4.** Associations between blood Pb, Hg, and Cd concentrations and HOMA-IR ^1^.

**Supplementary Table S5.** Associations between blood Pb, Hg, and Cd concentrations and the TyG index according to sex among participants with HOMA-IR values (n=3678).

**Supplementary Table S6.** Comparing current study with Moon SS’s study.

**Supplementary Table S7.** The limits of detection (LOD) for blood Pb, Hg, and Cd.

**
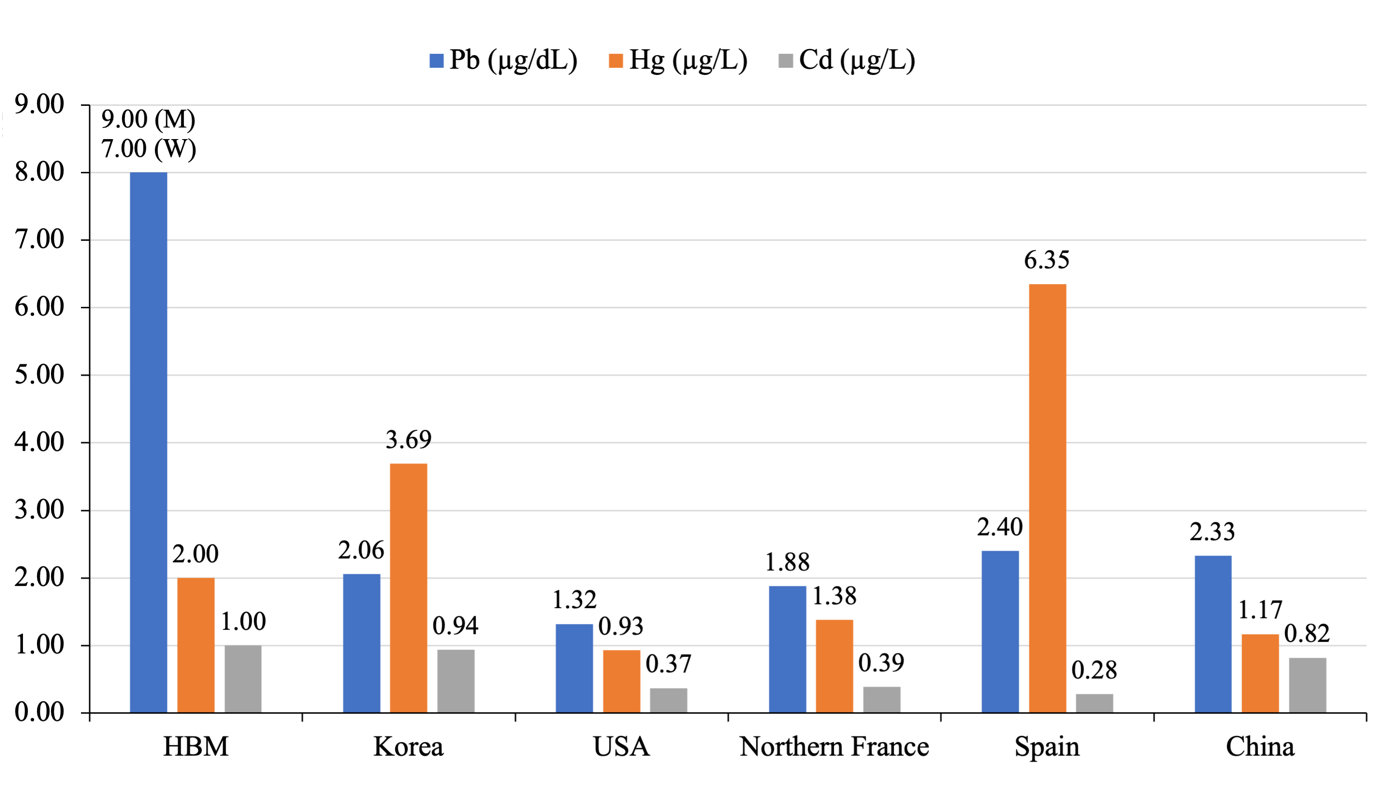
 Supplementary Figure S1.** The reference values of blood Pb, Hg, and Cd concentrations recommended by the HBM Commission and the concentrations in Korea, USA, Northern France, Spain, and China. Pb, lead; Hg, mercury; Cd, cadmium; HBM, Human Biomonitoring. References: HBM, [41]; USA, [42]; Northern France, [43]; Spain, [44, 45, 48]; and China, [46].

**Supplementary Table S1.** Associations between blood Pb concentrations and the TyG index according to sex (for the cut-off points 2 to 6).

|  |  | **Overall** | | | | **Men** | | | | **Women** | | | |
| --- | --- | --- | --- | --- | --- | --- | --- | --- | --- | --- | --- | --- | --- |
|  |  | **Case/total** | **OR (95% CI) ^6^** | **OR (95% CI) ^7^** | **OR (95% CI) ^8^** | **Case/total** | **OR (95% CI) ^6^** | **OR (95% CI) ^7^** | **OR (95% CI) ^8^** | **Case/total** | **OR (95% CI) ^6^** | **OR (95% CI) ^7^** | **OR (95% CI) ^8^** |
| Cut-off point 2 ^1^ | |  |  |  |  |  |  |  |  |  |  |  |  |
|  | Continuous | 1,709/9,645 | 2.44 (2.13, 2.81) | 1.25 (1.04, 1.51) | 1.23 (1.02, 1.48) | 1,151/4,314 | 1.94 (1.62, 2.33) | 1.42 (1.12, 1.80) | 1.35 (1.06, 1.72) | 558/5,331 | 1.65 (1.32, 2.08) | 1.07 (0.81, 1.42) | 1.13 (0.85, 1.49) |
|  | Quartile 1 | 234/2,411 | 1.00 (Reference) | 1.00 (Reference) | 1.00 (Reference) | 86/523 | 1.00 (Reference) | 1.00 (Reference) | 1.00 (Reference) | 148/1,888 | 1.00 (Reference) | 1.00 (Reference) | 1.00 (Reference) |
|  | Quartile 2 | 340/2,410 | 1.62 (1.30, 2.03) | 1.26 (0.99, 1.62) | 1.25 (0.98, 1.61) | 193/886 | 1.76 (1.26, 2.44) | 1.62 (1.12, 2.35) | 1.59 (1.10, 2.30) | 147/1,524 | 1.25 (0.92, 1.70) | 1.07 (0.78, 1.47) | 1.11 (0.81, 1.52) |
|  | Quartile 3 | 482/2,412 | 2.38 (1.94, 2.91) | 1.41 (1.11, 1.80) | 1.39 (1.10, 1.77) | 328/1,245 | 2.20 (1.63, 2.95) | 1.79 (1.25, 2.55) | 1.72 (1.20, 2.46) | 154/1,167 | 1.80 (1.32, 2.45) | 1.20 (0.86, 1.68) | 1.26 (0.90, 1.76) |
|  | Quartile 4 | 653/2,412 | 3.33 (2.73, 4.07) | 1.46 (1.13, 1.90) | 1.44 (1.11, 1.86) | 544/1,660 | 2.89 (2.16, 3.86) | 2.06 (1.42, 2.99) | 1.94 (1.33, 2.83) | 109/752 | 1.70 (1.24, 2.33) | 1.03 (0.70, 1.51) | 1.09 (0.74, 1.61) |
|  | *p* for trend |  | <.001 | 0.004 | 0.007 |  | <.001 | <.001 | 0.002 |  | <.001 | 0.613 | 0.402 |
| Cut-off point 3 ^2^ | |  |  |  |  |  |  |  |  |  |  |  |  |
|  | Continuous | 5,564/9,645 | 1.57 (1.41, 1.75) | 1.09 (0.95, 1.25) | 1.07 (0.93, 1.23) | 2,502/4,314 | 1.94 (1.63, 2.31) | 1.26 (1.02, 1.56) | 1.21 (0.98, 1.49) | 3,062/5,331 | 1.51 (1.30, 1.75) | 0.98 (0.82, 1.18) | 0.98 (0.82, 1.18) |
|  | Quartile 1 | 1,220/2,411 | 1.00 (Reference) | 1.00 (Reference) | 1.00 (Reference) | 228/523 | 1.00 (Reference) | 1.00 (Reference) | 1.00 (Reference) | 992/1,888 | 1.00 (Reference) | 1.00 (Reference) | 1.00 (Reference) |
|  | Quartile 2 | 1,294/2,410 | 1.23 (1.06, 1.42) | 1.06 (0.90, 1.24) | 1.04 (0.89, 1.22) | 462/886 | 1.76 (1.35, 2.29) | 1.52 (1.14, 2.04) | 1.48 (1.10, 1.99) | 832/1,524 | 1.11 (0.94, 1.31) | 0.90 (0.75, 1.09) | 0.91 (0.75, 1.10) |
|  | Quartile 3 | 1,472/2,412 | 1.60 (1.39, 1.84) | 1.18 (1.00, 1.38) | 1.15 (0.98, 1.35) | 728/1,245 | 2.09 (1.64, 2.68) | 1.48 (1.11, 1.97) | 1.42 (1.06, 1.90) | 744/1,167 | 1.63 (1.37, 1.94) | 1.09 (0.88, 1.34) | 1.08 (0.88, 1.33) |
|  | Quartile 4 | 1,578/2,412 | 1.79 (1.55, 2.07) | 1.16 (0.97, 1.40) | 1.14 (0.94, 1.37) | 1,084/1,660 | 2.70 (2.12, 3.45) | 1.64 (1.21, 2.23) | 1.55 (1.14, 2.10) | 494/752 | 1.55 (1.26, 1.91) | 0.94 (0.73, 1.22) | 0.94 (0.73, 1.22) |
|  | *p* for trend |  | <.001 | 0.064 | 0.115 |  | <.001 | 0.008 | 0.025 |  | <.001 | 0.994 | 0.992 |
| Cut-off point 4 ^3^ | |  |  |  |  |  |  |  |  |  |  |  |  |
|  | Continuous | 2,755/9,645 | 2.38 (2.10, 2.70) | 1.21 (1.03, 1.41) | 1.18 (1.01, 1.38) | 1,763/4,314 | 1.96 (1.65, 2.32) | 1.37 (1.10, 1.70) | 1.30 (1.04, 1.61) | 992/5,331 | 1.57 (1.31, 1.88) | 1.04 (0.83, 1.29) | 1.08 (0.86, 1.35) |
|  | Quartile 1 | 445/2,411 | 1.00 (Reference) | 1.00 (Reference) | 1.00 (Reference) | 153/523 | 1.00 (Reference) | 1.00 (Reference) | 1.00 (Reference) | 292/1,888 | 1.00 (Reference) | 1.00 (Reference) | 1.00 (Reference) |
|  | Quartile 2 | 549/2,410 | 1.38 (1.16, 1.64) | 1.03 (0.85, 1.26) | 1.02 (0.84, 1.25) | 297/886 | 1.48 (1.12, 1.95) | 1.30 (0.95, 1.78) | 1.26 (0.92, 1.73) | 252/1,524 | 1.09 (0.87, 1.37) | 0.91 (0.71, 1.17) | 0.94 (0.73, 1.20) |
|  | Quartile 3 | 772/2,412 | 2.06 (1.75, 2.42) | 1.18 (0.97, 1.44) | 1.16 (0.95, 1.41) | 517/1,245 | 2.02 (1.57, 2.60) | 1.54 (1.14, 2.09) | 1.46 (1.08, 1.99) | 255/1,167 | 1.45 (1.15, 1.84) | 0.95 (0.72, 1.24) | 0.98 (0.75, 1.29) |
|  | Quartile 4 | 989/2,412 | 2.94 (2.51, 3.45) | 1.26 (1.03, 1.55) | 1.23 (1.00, 1.51) | 796/1,660 | 2.55 (1.99, 3.26) | 1.67 (1.22, 2.29) | 1.55 (1.13, 2.13) | 193/752 | 1.64 (1.29, 2.10) | 1.02 (0.75, 1.38) | 1.07 (0.79, 1.45) |
|  | *p* for trend |  | <.001 | 0.012 | 0.026 |  | <.001 | 0.001 | 0.007 |  | <.001 | 0.999 | 0.753 |
| Cut-off point 5 ^4^ | |  |  |  |  |  |  |  |  |  |  |  |  |
|  | Continuous | 4,077/9,645 | 2.25 (2.00, 2.53) | 1.11 (0.95, 1.28) | 1.09 (0.94, 1.26) | 2,432/4,314 | 1.88 (1.58, 2.24) | 1.22 (0.98, 1.51) | 1.16 (0.94, 1.44) | 1,645/5,331 | 1.57 (1.35, 1.83) | 1.02 (0.84, 1.23) | 1.03 (0.85, 1.24) |
|  | Quartile 1 | 718/2,411 | 1.00 (Reference) | 1.00 (Reference) | 1.00 (Reference) | 224/523 | 1.00 (Reference) | 1.00 (Reference) | 1.00 (Reference) | 494/1,888 | 1.00 (Reference) | 1.00 (Reference) | 1.00 (Reference) |
|  | Quartile 2 | 878/2,410 | 1.47 (1.27, 1.71) | 1.12 (0.95, 1.33) | 1.11 (0.94, 1.31) | 446/886 | 1.69 (1.30, 2.20) | 1.45 (1.09, 1.94) | 1.41 (1.05, 1.89) | 432/1,524 | 1.18 (0.98, 1.41) | 0.98 (0.80, 1.20) | 0.99 (0.81, 1.22) |
|  | Quartile 3 | 1,124/2,412 | 2.07 (1.80, 2.39) | 1.18 (1.00, 1.39) | 1.15 (0.98, 1.36) | 706/1,245 | 2.00 (1.57, 2.55) | 1.40 (1.05, 1.86) | 1.33 (1.00, 1.78) | 418/1,167 | 1.62 (1.35, 1.95) | 1.07 (0.86, 1.33) | 1.08 (0.87, 1.34) |
|  | Quartile 4 | 1,357/2,412 | 2.86 (2.48, 3.30) | 1.22 (1.01, 1.47) | 1.19 (0.98, 1.44) | 1,056/1,660 | 2.59 (2.03, 3.29) | 1.56 (1.15, 2.11) | 1.46 (1.08, 1.97) | 301/752 | 1.70 (1.39, 2.08) | 1.05 (0.81, 1.35) | 1.06 (0.82, 1.38) |
|  | *p* for trend |  | <.001 | 0.036 | 0.067 |  | <.001 | 0.017 | 0.054 |  | <.001 | 0.585 | 0.503 |
| Cut-off point 6 ^5^ | |  |  |  |  |  |  |  |  |  |  |  |  |
|  | Continuous | 682/9,645 | 2.55 (2.09, 3.11) | 1.24 (0.93, 1.64) | 1.20 (0.90, 1.60) | 496/4,314 | 1.97 (1.53, 2.53) | 1.28 (0.91, 1.80) | 1.22 (0.86, 1.72) | 186/5,331 | 1.57 (1.08, 2.29) | 1.15 (0.72, 1.85) | 1.23 (0.77, 1.95) |
|  | Quartile 1 | 77/2,411 | 1.00 (Reference) | 1.00 (Reference) | 1.00 (Reference) | 28/523 | 1.00 (Reference) | 1.00 (Reference) | 1.00 (Reference) | 49/1,888 | 1.00 (Reference) | 1.00 (Reference) | 1.00 (Reference) |
|  | Quartile 2 | 131/2,410 | 1.88 (1.31, 2.69) | 1.43 (0.96, 2.11) | 1.40 (0.95, 2.08) | 86/886 | 2.53 (1.46, 4.38) | 2.33 (1.27, 4.26) | 2.29 (1.25, 4.20) | 45/1,524 | 1.11 (0.67, 1.85) | 0.97 (0.57, 1.66) | 1.01 (0.59, 1.74) |
|  | Quartile 3 | 184/2,412 | 2.59 (1.83, 3.67) | 1.46 (0.98, 2.18) | 1.41 (0.95, 2.11) | 133/1,245 | 2.82 (1.67, 4.76) | 2.17 (1.19, 3.95) | 2.08 (1.13, 3.80) | 51/1,167 | 1.61 (0.96, 2.71) | 1.11 (0.63, 1.95) | 1.18 (0.67, 2.07) |
|  | Quartile 4 | 290/2,412 | 4.09 (2.98, 5.61) | 1.68 (1.12, 2.50) | 1.61 (1.08, 2.41) | 249/1,660 | 4.00 (2.43, 6.59) | 2.59 (1.42, 4.71) | 2.43 (1.33, 4.46) | 41/752 | 1.67 (1.01, 2.75) | 1.16 (0.65, 2.08) | 1.24 (0.69, 2.24) |
|  | *p* for trend |  | <.001 | 0.016 | 0.028 |  | <.001 | 0.010 | 0.023 |  | 0.021 | 0.574 | 0.417 |

Pb, lead; TyG index, triglyceride glucose index; OR, odds ratio; CI, confidence interval.

^1^ The cut-off point 2: 9.03 for incident cardiovascular disease proposed in an Iranian study.

^2^ The cut-off point 3: 8.49 in men and 8.12 in women for insulin resistance proposed in a Japanese study.

^3^ The cut-off point 4: 8.76 for insulin resistance proposed in a Chinese study.

^4^ The cut-off point 5: 8.52 for metabolic syndrome proposed in a Korean study.

^5^ The cut-off point 6: 9.44 calculated according to the American Diabetes Association and the Third Report of the Expert Panel on Detection, Evaluation, and Treatment of High Blood Cholesterol in Adults by the National Cholesterol Education Program criteria for type 2 diabetes (fasting glucose ≥126 mg/dL) and hypertriglyceridemia (triglyceride ≥200 mg/dL).

^6^ Model 1, with no adjustment.

^7^ Model 2, adjusted for age, sex (for men and women combined), survey year, BMI, alcohol consumption, smoking status, educational level, occupation, physical activity, menopausal status, grain consumption, fish consumption, seaweed consumption, vegetable consumption, and mushroom consumption.

^8^ Model 3, further adjusted for natural log–transformed blood concentrations of Hg and Cd.

**Supplementary Table S2.** Associations between blood Hg concentrations and the TyG index according to sex (for the cut-off points 2 to 6).

|  |  | **Overall** | | | | **Men** | | | | **Women** | | | |
| --- | --- | --- | --- | --- | --- | --- | --- | --- | --- | --- | --- | --- | --- |
|  |  | **Case/total** | **OR (95% CI) ^6^** | **OR (95% CI) ^7^** | **OR (95% CI) ^8^** | **Case/total** | **OR (95% CI) ^6^** | **OR (95% CI) ^7^** | **OR (95% CI) ^8^** | **Case/total** | **OR (95% CI) ^6^** | **OR (95% CI) ^7^** | **OR (95% CI) ^8^** |
| Cut-off point 2 ^1^ | |  |  |  |  |  |  |  |  |  |  |  |  |
|  | Continuous | 1,709/9,645 | 1.66 (1.49, 1.84) | 0.98 (0.87, 1.10) | 0.96 (0.85, 1.08) | 1,151/4,314 | 1.56 (1.38, 1.77) | 1.06 (0.91, 1.22) | 1.02 (0.88, 1.18) | 558/5,331 | 1.06 (0.88, 1.29) | 0.84 (0.69, 1.03) | 0.84 (0.69, 1.04) |
|  | Quartile 1 | 289/2,411 | 1.00 (Reference) | 1.00 (Reference) | 1.00 (Reference) | 127/681 | 1.00 (Reference) | 1.00 (Reference) | 1.00 (Reference) | 162/1,730 | 1.00 (Reference) | 1.00 (Reference) | 1.00 (Reference) |
|  | Quartile 2 | 364/2,411 | 1.32 (1.09, 1.61) | 1.06 (0.86, 1.32) | 1.05 (0.84, 1.30) | 206/902 | 1.43 (1.08, 1.90) | 1.28 (0.93, 1.75) | 1.24 (0.90, 1.70) | 158/1,509 | 1.08 (0.81, 1.42) | 0.99 (0.74, 1.32) | 0.99 (0.74, 1.33) |
|  | Quartile 3 | 463/2,412 | 1.66 (1.38, 2.01) | 1.04 (0.83, 1.29) | 1.01 (0.81, 1.25) | 329/1,185 | 1.85 (1.42, 2.41) | 1.30 (0.96, 1.77) | 1.24 (0.91, 1.68) | 134/1,227 | 1.04 (0.77, 1.40) | 0.85 (0.62, 1.17) | 0.85 (0.61, 1.17) |
|  | Quartile 4 | 593/2,411 | 2.17 (1.82, 2.60) | 0.96 (0.77, 1.18) | 0.92 (0.74, 1.13) | 489/1,546 | 2.11 (1.64, 2.71) | 1.16 (0.86, 1.57) | 1.09 (0.80, 1.47) | 104/865 | 1.08 (0.79, 1.48) | 0.78 (0.56, 1.09) | 0.78 (0.56, 1.10) |
|  | *p* for trend |  | <.001 | 0.523 | 0.292 |  | <.001 | 0.708 | 0.913 |  | 0.655 | 0.115 | 0.117 |
| Cut-off point 3 ^2^ | |  |  |  |  |  |  |  |  |  |  |  |  |
|  | Continuous | 5,564/9,645 | 1.23 (1.13, 1.33) | 0.92 (0.84, 1.01) | 0.90 (0.82, 0.99) | 2,502/4,314 | 1.51 (1.35, 1.70) | 0.99 (0.87, 1.13) | 0.96 (0.84, 1.10) | 3,062/5,331 | 1.04 (0.93, 1.17) | 0.82 (0.72, 0.94) | 0.81 (0.71, 0.92) |
|  | Quartile 1 | 1,312/2,411 | 1.00 (Reference) | 1.00 (Reference) | 1.00 (Reference) | 338/681 | 1.00 (Reference) | 1.00 (Reference) | 1.00 (Reference) | 974/1,730 | 1.00 (Reference) | 1.00 (Reference) | 1.00 (Reference) |
|  | Quartile 2 | 1,312/2,411 | 0.96 (0.84, 1.10) | 0.84 (0.73, 0.97) | 0.82 (0.71, 0.95) | 478/902 | 1.15 (0.92, 1.44) | 0.95 (0.74, 1.21) | 0.92 (0.72, 1.18) | 834/1,509 | 0.91 (0.77, 1.07) | 0.80 (0.67, 0.96) | 0.79 (0.66, 0.95) |
|  | Quartile 3 | 1,446/2,412 | 1.20 (1.05, 1.37) | 0.91 (0.78, 1.05) | 0.88 (0.76, 1.03) | 710/1,185 | 1.57 (1.27, 1.95) | 0.99 (0.77, 1.27) | 0.95 (0.73, 1.22) | 736/1,227 | 1.07 (0.90, 1.26) | 0.86 (0.71, 1.05) | 0.85 (0.70, 1.04) |
|  | Quartile 4 | 1,494/2,411 | 1.22 (1.07, 1.40) | 0.76 (0.65, 0.89) | 0.73 (0.63, 0.86) | 976/1,546 | 1.73 (1.41, 2.12) | 0.85 (0.67, 1.09) | 0.81 (0.63, 1.04) | 518/865 | 0.95 (0.78, 1.17) | 0.65 (0.52, 0.82) | 0.64 (0.51, 0.80) |
|  | *p* for trend |  | <.001 | 0.003 | 0.001 |  | <.001 | 0.186 | 0.080 |  | 0.996 | 0.001 | <.001 |
| Cut-off point 4 ^3^ | |  |  |  |  |  |  |  |  |  |  |  |  |
|  | Continuous | 2,755/9,645 | 1.62 (1.48, 1.77) | 0.99 (0.90, 1.10) | 0.97 (0.88, 1.07) | 1,763/4,314 | 1.57 (1.39, 1.77) | 1.07 (0.93, 1.23) | 1.03 (0.90, 1.18) | 992/5,331 | 1.06 (0.92, 1.22) | 0.85 (0.72, 1.00) | 0.85 (0.72, 1.00) |
|  | Quartile 1 | 511/2,411 | 1.00 (Reference) | 1.00 (Reference) | 1.00 (Reference) | 212/681 | 1.00 (Reference) | 1.00 (Reference) | 1.00 (Reference) | 299/1,730 | 1.00 (Reference) | 1.00 (Reference) | 1.00 (Reference) |
|  | Quartile 2 | 597/2,411 | 1.24 (1.06, 1.45) | 1.00 (0.83, 1.19) | 0.98 (0.82, 1.17) | 319/902 | 1.30 (1.01, 1.67) | 1.14 (0.86, 1.51) | 1.10 (0.83, 1.46) | 278/1,509 | 1.06 (0.86, 1.30) | 0.95 (0.76, 1.20) | 0.96 (0.76, 1.20) |
|  | Quartile 3 | 738/2,412 | 1.56 (1.34, 1.83) | 0.99 (0.82, 1.19) | 0.96 (0.80, 1.15) | 507/1,185 | 1.75 (1.38, 2.22) | 1.20 (0.91, 1.58) | 1.13 (0.86, 1.50) | 231/1,227 | 1.02 (0.81, 1.28) | 0.82 (0.63, 1.06) | 0.82 (0.63, 1.07) |
|  | Quartile 4 | 909/2,411 | 2.07 (1.79, 2.40) | 0.96 (0.81, 1.15) | 0.93 (0.77, 1.11) | 725/1,546 | 2.02 (1.61, 2.52) | 1.11 (0.85, 1.45) | 1.03 (0.79, 1.36) | 184/865 | 1.10 (0.86, 1.40) | 0.81 (0.62, 1.07) | 0.81 (0.61, 1.08) |
|  | *p* for trend |  | <.001 | 0.652 | 0.383 |  | <.001 | 0.618 | 0.977 |  | 0.548 | 0.085 | 0.091 |
| Cut-off point 5 ^4^ | |  |  |  |  |  |  |  |  |  |  |  |  |
|  | Continuous | 4,077/9,645 | 1.56 (1.44, 1.69) | 0.96 (0.88, 1.06) | 0.95 (0.86, 1.04) | 2,432/4,314 | 1.53 (1.37, 1.71) | 1.00 (0.88, 1.14) | 0.97 (0.86, 1.11) | 1,645/5,331 | 1.12 (0.99, 1.26) | 0.89 (0.78, 1.02) | 0.89 (0.77, 1.02) |
|  | Quartile 1 | 825/2,411 | 1.00 (Reference) | 1.00 (Reference) | 1.00 (Reference) | 324/681 | 1.00 (Reference) | 1.00 (Reference) | 1.00 (Reference) | 501/1,730 | 1.00 (Reference) | 1.00 (Reference) | 1.00 (Reference) |
|  | Quartile 2 | 908/2,411 | 1.10 (0.96, 1.25) | 0.86 (0.74, 1.00) | 0.85 (0.73, 0.99) | 464/902 | 1.17 (0.93, 1.47) | 0.96 (0.75, 1.23) | 0.94 (0.73, 1.20) | 444/1,509 | 0.94 (0.79, 1.11) | 0.82 (0.67, 0.99) | 0.81 (0.67, 0.99) |
|  | Quartile 3 | 1,077/2,412 | 1.43 (1.25, 1.64) | 0.89 (0.76, 1.05) | 0.87 (0.74, 1.02) | 683/1,185 | 1.56 (1.26, 1.94) | 0.99 (0.77, 1.27) | 0.94 (0.73, 1.21) | 394/1,227 | 1.04 (0.86, 1.25) | 0.82 (0.66, 1.02) | 0.81 (0.65, 1.01) |
|  | Quartile 4 | 1,267/2,411 | 1.86 (1.63, 2.13) | 0.86 (0.73, 1.01) | 0.83 (0.71, 0.98) | 961/1,546 | 1.81 (1.48, 2.22) | 0.91 (0.71, 1.16) | 0.86 (0.67, 1.10) | 306/865 | 1.12 (0.92, 1.36) | 0.80 (0.63, 1.00) | 0.79 (0.63, 0.99) |
|  | *p* for trend |  | <.001 | 0.103 | 0.046 |  | <.001 | 0.435 | 0.232 |  | 0.260 | 0.039 | 0.033 |
| Cut-off point 6 ^5^ | |  |  |  |  |  |  |  |  |  |  |  |  |
|  | Continuous | 682/9,645 | 1.84 (1.59, 2.12) | 1.05 (0.89, 1.23) | 1.02 (0.87, 1.20) | 496/4,314 | 1.65 (1.40, 1.95) | 1.11 (0.91, 1.35) | 1.08 (0.88, 1.31) | 186/5,331 | 1.09 (0.84, 1.43) | 0.90 (0.68, 1.19) | 0.90 (0.68, 1.20) |
|  | Quartile 1 | 83/2,411 | 1.00 (Reference) | 1.00 (Reference) | 1.00 (Reference) | 37/681 | 1.00 (Reference) | 1.00 (Reference) | 1.00 (Reference) | 46/1,730 | 1.00 (Reference) | 1.00 (Reference) | 1.00 (Reference) |
|  | Quartile 2 | 154/2,411 | 1.96 (1.40, 2.74) | 1.57 (1.10, 2.24) | 1.55 (1.08, 2.21) | 93/902 | 2.37 (1.48, 3.79) | 2.24 (1.35, 3.71) | 2.20 (1.33, 3.64) | 61/1,509 | 1.38 (0.85, 2.26) | 1.29 (0.78, 2.12) | 1.28 (0.78, 2.12) |
|  | Quartile 3 | 187/2,412 | 2.19 (1.59, 3.02) | 1.32 (0.93, 1.88) | 1.29 (0.91, 1.83) | 139/1,185 | 2.57 (1.66, 3.97) | 1.86 (1.15, 3.03) | 1.81 (1.12, 2.93) | 48/1,227 | 1.23 (0.74, 2.05) | 1.07 (0.62, 1.85) | 1.06 (0.61, 1.84) |
|  | Quartile 4 | 258/2,411 | 3.13 (2.29, 4.28) | 1.32 (0.93, 1.87) | 1.27 (0.89, 1.80) | 227/1,546 | 3.40 (2.21, 5.21) | 1.94 (1.19, 3.16) | 1.85 (1.13, 3.01) | 31/865 | 0.95 (0.54, 1.68) | 0.70 (0.39, 1.26) | 0.70 (0.39, 1.25) |
|  | *p* for trend |  | <.001 | 0.438 | 0.619 |  | <.001 | 0.115 | 0.193 |  | 0.966 | 0.263 | 0.257 |

Pb, lead; TyG index, triglyceride glucose index; OR, odds ratio; CI, confidence interval.

^1^ The cut-off point 2: 9.03 for incident cardiovascular disease proposed in an Iranian study.

^2^ The cut-off point 3: 8.49 in men and 8.12 in women for insulin resistance proposed in a Japanese study.

^3^ The cut-off point 4: 8.76 for insulin resistance proposed in a Chinese study.

^4^ The cut-off point 5: 8.52 for metabolic syndrome proposed in a Korean study.

^5^ The cut-off point 6: 9.44 calculated according to the American Diabetes Association and the Third Report of the Expert Panel on Detection, Evaluation, and Treatment of High Blood Cholesterol in Adults by the National Cholesterol Education Program criteria for type 2 diabetes (fasting glucose ≥126 mg/dL) and hypertriglyceridemia (triglyceride ≥200 mg/dL).

^6^ Model 1, with no adjustment.

^7^ Model 2, adjusted for age, sex (for men and women combined), survey year, BMI, alcohol consumption, smoking status, educational level, occupation, physical activity, menopausal status, grain consumption, fish consumption, seaweed consumption, vegetable consumption, and mushroom consumption.

^8^ Model 3, further adjusted for natural log–transformed blood concentrations of Pb and Cd.

**Supplementary Table S3.** Associations between blood Cd concentrations and the TyG index according to sex (for the cut-off points 2 to 6).

|  |  | **Overall** | | | | **Men** | | | | **Women** | | | |
| --- | --- | --- | --- | --- | --- | --- | --- | --- | --- | --- | --- | --- | --- |
|  |  | **Case/total** | **OR (95% CI) ^6^** | **OR (95% CI) ^7^** | **OR (95% CI) ^8^** | **Case/total** | **OR (95% CI) ^6^** | **OR (95% CI) ^7^** | **OR (95% CI) ^8^** | **Case/total** | **OR (95% CI) ^6^** | **OR (95% CI) ^7^** | **OR (95% CI) ^8^** |
| Cut-off point 2 ^1^ | |  |  |  |  |  |  |  |  |  |  |  |  |
|  | Continuous | 1,709/9,645 | 1.42 (1.28, 1.57) | 1.15 (1.00, 1.32) | 1.12 (0.97, 1.30) | 1,151/4,314 | 1.55 (1.37, 1.75) | 1.31 (1.10, 1.58) | 1.27 (1.06, 1.53) | 558/5,331 | 1.65 (1.36, 2.01) | 0.91 (0.71, 1.15) | 0.91 (0.72, 1.16) |
|  | Quartile 1 | 326/2,412 | 1.00 (Reference) | 1.00 (Reference) | 1.00 (Reference) | 240/1,232 | 1.00 (Reference) | 1.00 (Reference) | 1.00 (Reference) | 86/1,180 | 1.00 (Reference) | 1.00 (Reference) | 1.00 (Reference) |
|  | Quartile 2 | 400/2,412 | 1.27 (1.05, 1.53) | 1.00 (0.81, 1.23) | 0.98 (0.79, 1.21) | 280/1,104 | 1.41 (1.12, 1.76) | 1.10 (0.85, 1.42) | 1.07 (0.82, 1.38) | 120/1,308 | 1.28 (0.89, 1.82) | 0.82 (0.57, 1.19) | 0.83 (0.57, 1.19) |
|  | Quartile 3 | 460/2,408 | 1.45 (1.21, 1.74) | 1.01 (0.80, 1.26) | 0.98 (0.78, 1.23) | 312/1,017 | 1.69 (1.35, 2.12) | 1.17 (0.89, 1.54) | 1.12 (0.85, 1.48) | 148/1,391 | 1.58 (1.13, 2.21) | 0.73 (0.50, 1.06) | 0.73 (0.50, 1.05) |
|  | Quartile 4 | 523/2,413 | 1.75 (1.47, 2.09) | 1.22 (0.96, 1.55) | 1.18 (0.93, 1.50) | 319/961 | 2.04 (1.63, 2.54) | 1.56 (1.15, 2.12) | 1.47 (1.08, 2.00) | 204/1,452 | 2.14 (1.57, 2.92) | 0.78 (0.53, 1.15) | 0.78 (0.53, 1.15) |
|  | *p* for trend |  | <.001 | 0.101 | 0.174 |  | <.001 | 0.006 | 0.019 |  | <.001 | 0.279 | 0.276 |
| Cut-off point 3 ^2^ | |  |  |  |  |  |  |  |  |  |  |  |  |
|  | Continuous | 5,564/9,645 | 1.68 (1.55, 1.82) | 1.19 (1.07, 1.32) | 1.19 (1.07, 1.33) | 2,502/4,314 | 1.60 (1.43, 1.80) | 1.30 (1.11, 1.53) | 1.28 (1.09, 1.50) | 3,062/5,331 | 1.77 (1.58, 1.98) | 1.11 (0.96, 1.29) | 1.14 (0.98, 1.33) |
|  | Quartile 1 | 1,119/2,412 | 1.00 (Reference) | 1.00 (Reference) | 1.00 (Reference) | 579/1,232 | 1.00 (Reference) | 1.00 (Reference) | 1.00 (Reference) | 540/1,180 | 1.00 (Reference) | 1.00 (Reference) | 1.00 (Reference) |
|  | Quartile 2 | 1,328/2,412 | 1.35 (1.19, 1.53) | 0.98 (0.85, 1.13) | 0.98 (0.85, 1.13) | 644/1,104 | 1.57 (1.30, 1.89) | 1.16 (0.93, 1.44) | 1.15 (0.92, 1.42) | 684/1,308 | 1.17 (0.97, 1.41) | 0.84 (0.69, 1.03) | 0.86 (0.70, 1.05) |
|  | Quartile 3 | 1,490/2,408 | 1.72 (1.51, 1.97) | 1.01 (0.86, 1.18) | 1.01 (0.86, 1.18) | 645/1,017 | 1.86 (1.53, 2.27) | 1.20 (0.93, 1.54) | 1.17 (0.91, 1.51) | 845/1,391 | 1.60 (1.33, 1.93) | 0.84 (0.68, 1.05) | 0.86 (0.69, 1.07) |
|  | Quartile 4 | 1,627/2,413 | 2.29 (1.99, 2.62) | 1.28 (1.08, 1.53) | 1.29 (1.08, 1.54) | 634/961 | 2.11 (1.72, 2.59) | 1.59 (1.21, 2.10) | 1.55 (1.18, 2.04) | 993/1,452 | 2.38 (1.98, 2.86) | 1.09 (0.86, 1.40) | 1.14 (0.89, 1.46) |
|  | *p* for trend |  | <.001 | 0.008 | 0.007 |  | <.001 | 0.002 | 0.004 |  | <.001 | 0.416 | 0.254 |
| Cut-off point 4 ^3^ | |  |  |  |  |  |  |  |  |  |  |  |  |
|  | Continuous | 2,755/9,645 | 1.41 (1.29, 1.54) | 1.15 (1.01, 1.30) | 1.13 (1.00, 1.28) | 1,763/4,314 | 1.59 (1.41, 1.78) | 1.36 (1.15, 1.61) | 1.32 (1.12, 1.57) | 992/5,331 | 1.59 (1.36, 1.86) | 0.93 (0.76, 1.13) | 0.94 (0.77, 1.14) |
|  | Quartile 1 | 532/2,412 | 1.00 (Reference) | 1.00 (Reference) | 1.00 (Reference) | 384/1,232 | 1.00 (Reference) | 1.00 (Reference) | 1.00 (Reference) | 148/1,180 | 1.00 (Reference) | 1.00 (Reference) | 1.00 (Reference) |
|  | Quartile 2 | 658/2,412 | 1.30 (1.11, 1.51) | 1.03 (0.86, 1.24) | 1.02 (0.85, 1.22) | 434/1,104 | 1.40 (1.14, 1.70) | 1.06 (0.85, 1.34) | 1.04 (0.82, 1.31) | 224/1,308 | 1.42 (1.08, 1.85) | 1.01 (0.75, 1.36) | 1.02 (0.76, 1.38) |
|  | Quartile 3 | 734/2,408 | 1.48 (1.27, 1.73) | 1.04 (0.86, 1.26) | 1.02 (0.84, 1.24) | 469/1,017 | 1.78 (1.45, 2.19) | 1.23 (0.95, 1.59) | 1.18 (0.91, 1.53) | 265/1,391 | 1.61 (1.25, 2.07) | 0.82 (0.61, 1.12) | 0.83 (0.61, 1.12) |
|  | Quartile 4 | 831/2,413 | 1.76 (1.52, 2.05) | 1.25 (1.02, 1.53) | 1.21 (0.99, 1.49) | 476/961 | 2.08 (1.70, 2.55) | 1.66 (1.26, 2.19) | 1.58 (1.20, 2.08) | 355/1,452 | 2.18 (1.71, 2.79) | 0.91 (0.66, 1.26) | 0.93 (0.67, 1.28) |
|  | *p* for trend |  | <.001 | 0.037 | 0.069 |  | <.001 | <.001 | 0.002 |  | <.001 | 0.453 | 0.507 |
| Cut-off point 5 ^4^ | |  |  |  |  |  |  |  |  |  |  |  |  |
|  | Continuous | 4,077/9,645 | 1.45 (1.34, 1.58) | 1.15 (1.03, 1.29) | 1.15 (1.03, 1.29) | 2,432/4,314 | 1.59 (1.42, 1.79) | 1.32 (1.12, 1.55) | 1.30 (1.11, 1.53) | 1,645/5,331 | 1.68 (1.48, 1.91) | 1.01 (0.86, 1.19) | 1.03 (0.87, 1.21) |
|  | Quartile 1 | 818/2,412 | 1.00 (Reference) | 1.00 (Reference) | 1.00 (Reference) | 558/1,232 | 1.00 (Reference) | 1.00 (Reference) | 1.00 (Reference) | 260/1,180 | 1.00 (Reference) | 1.00 (Reference) | 1.00 (Reference) |
|  | Quartile 2 | 991/2,412 | 1.33 (1.16, 1.52) | 1.04 (0.89, 1.22) | 1.04 (0.89, 1.22) | 625/1,104 | 1.54 (1.27, 1.86) | 1.15 (0.92, 1.43) | 1.14 (0.91, 1.42) | 366/1,308 | 1.33 (1.08, 1.64) | 0.94 (0.74, 1.20) | 0.95 (0.75, 1.21) |
|  | Quartile 3 | 1,075/2,408 | 1.50 (1.31, 1.73) | 1.02 (0.86, 1.22) | 1.01 (0.85, 1.21) | 633/1,017 | 1.89 (1.55, 2.30) | 1.26 (0.98, 1.61) | 1.24 (0.96, 1.58) | 442/1,391 | 1.57 (1.28, 1.92) | 0.81 (0.63, 1.04) | 0.81 (0.63, 1.04) |
|  | Quartile 4 | 1,193/2,413 | 1.85 (1.61, 2.13) | 1.26 (1.04, 1.51) | 1.25 (1.03, 1.50) | 616/961 | 2.07 (1.69, 2.54) | 1.62 (1.23, 2.13) | 1.58 (1.20, 2.08) | 577/1,452 | 2.30 (1.88, 2.81) | 0.99 (0.76, 1.29) | 1.01 (0.77, 1.32) |
|  | *p* for trend |  | <.001 | 0.025 | 0.031 |  | <.001 | 0.001 | 0.001 |  | <.001 | 0.938 | 0.972 |
| Cut-off point 6 ^5^ | |  |  |  |  |  |  |  |  |  |  |  |  |
|  | Continuous | 682/9,645 | 1.50 (1.29, 1.74) | 1.19 (0.96, 1.47) | 1.16 (0.93, 1.44) | 496/4,314 | 1.73 (1.46, 2.04) | 1.28 (1.00, 1.66) | 1.25 (0.96, 1.62) | 186/5,331 | 1.51 (1.05, 2.17) | 0.87 (0.56, 1.33) | 0.84 (0.54, 1.31) |
|  | Quartile 1 | 111/2,412 | 1.00 (Reference) | 1.00 (Reference) | 1.00 (Reference) | 83/1,232 | 1.00 (Reference) | 1.00 (Reference) | 1.00 (Reference) | 28/1,180 | 1.00 (Reference) | 1.00 (Reference) | 1.00 (Reference) |
|  | Quartile 2 | 159/2,412 | 1.60 (1.19, 2.13) | 1.28 (0.92, 1.79) | 1.26 (0.90, 1.76) | 120/1,104 | 1.82 (1.30, 2.56) | 1.33 (0.89, 1.99) | 1.30 (0.87, 1.94) | 39/1,308 | 1.42 (0.80, 2.53) | 1.07 (0.57, 2.00) | 1.05 (0.56, 1.99) |
|  | Quartile 3 | 198/2,408 | 1.80 (1.36, 2.40) | 1.27 (0.89, 1.79) | 1.23 (0.87, 1.74) | 144/1,017 | 2.30 (1.65, 3.21) | 1.39 (0.92, 2.10) | 1.34 (0.88, 2.03) | 54/1,391 | 1.49 (0.85, 2.62) | 0.84 (0.44, 1.61) | 0.82 (0.44, 1.55) |
|  | Quartile 4 | 214/2,413 | 2.09 (1.59, 2.74) | 1.47 (1.03, 2.10) | 1.42 (0.99, 2.03) | 149/961 | 2.61 (1.91, 3.57) | 1.60 (1.03, 2.47) | 1.53 (0.99, 2.37) | 65/1,452 | 2.16 (1.25, 3.72) | 0.94 (0.50, 1.79) | 0.92 (0.48, 1.74) |
|  | *p* for trend |  | <.001 | 0.047 | 0.077 |  | <.001 | 0.040 | 0.066 |  | 0.007 | 0.738 | 0.669 |

Pb, lead; TyG index, triglyceride glucose index; OR, odds ratio; CI, confidence interval.

^1^ The cut-off point 2: 9.03 for incident cardiovascular disease proposed in an Iranian study.

^2^ The cut-off point 3: 8.49 in men and 8.12 in women for insulin resistance proposed in a Japanese study.

^3^ The cut-off point 4: 8.76 for insulin resistance proposed in a Chinese study.

^4^ The cut-off point 5: 8.52 for metabolic syndrome proposed in a Korean study.

^5^ The cut-off point 6: 9.44 calculated according to the American Diabetes Association and the Third Report of the Expert Panel on Detection, Evaluation, and Treatment of High Blood Cholesterol in Adults by the National Cholesterol Education Program criteria for type 2 diabetes (fasting glucose ≥126 mg/dL) and hypertriglyceridemia (triglyceride ≥200 mg/dL).

^6^ Model 1, with no adjustment.

^7^ Model 2, adjusted for age, sex (for men and women combined), survey year, BMI, alcohol consumption, smoking status, educational level, occupation, physical activity, menopausal status, grain consumption, fish consumption, seaweed consumption, vegetable consumption, and mushroom consumption.

^8^ Model 3, further adjusted for natural log–transformed blood concentrations of Pb and Hg.

**Supplementary Table S4.** Associations between blood Pb, Hg, and Cd concentrations and HOMA-IR ^1^.

|  |  | **Case/total** | **OR (95% CI) ^2^** | **OR (95% CI) ^3^** | **OR (95% CI) ^4^** |
| --- | --- | --- | --- | --- | --- |
| Pb |  |  |  |  |  |
|  | Continuous | 1047/3678 | 1.17 (0.98, 1.39) | 1.00 (0.80, 1.26) | 0.98 (0.78, 1.24) |
|  | Quartile 1 | 185/697 | 1.00 (Reference) | 1.00 (Reference) | 1.00 (Reference) |
|  | Quartile 2 | 264/934 | 1.28 (0.98, 1.67) | 1.28 (0.96, 1.71) | 1.27 (0.95, 1.70) |
|  | Quartile 3 | 301/1019 | 1.40 (1.08, 1.81) | 1.19 (0.89, 1.59) | 1.17 (0.87, 1.57) |
|  | Quartile 4 | 297/1028 | 1.19 (0.93, 1.52) | 0.99 (0.73, 1.34) | 0.97 (0.71, 1.32) |
|  | *p* for trend |  | 0.196 | 0.62 | 0.514 |
| Hg |  |  |  |  |  |
|  | Continuous | 1047/3678 | 1.27 (1.07, 1.50) | 1.11 (0.91, 1.36) | 1.11 (0.91, 1.36) |
|  | Quartile 1 | 149/632 | 1.00 (Reference) | 1.00 (Reference) | 1.00 (Reference) |
|  | Quartile 2 | 252/919 | 1.16 (0.87, 1.55) | 1.19 (0.87, 1.63) | 1.19 (0.87, 1.63) |
|  | Quartile 3 | 308/1055 | 1.32 (1.00, 1.75) | 1.28 (0.94, 1.74) | 1.29 (0.95, 1.75) |
|  | Quartile 4 | 338/1072 | 1.44 (1.10, 1.90) | 1.24 (0.91, 1.70) | 1.25 (0.91, 1.70) |
|  | *p* for trend |  | 0.005 | 0.216 | 0.206 |
| Cd |  |  |  |  |  |
|  | Continuous | 1047/3678 | 1.04 (0.91, 1.20) | 1.01 (0.82, 1.23) | 1.00 (0.82, 1.22) |
|  | Quartile 1 | 273/944 | 1.00 (Reference) | 1.00 (Reference) | 1.00 (Reference) |
|  | Quartile 2 | 260/974 | 0.89 (0.70, 1.12) | 0.83 (0.64, 1.08) | 0.82 (0.63, 1.07) |
|  | Quartile 3 | 274/937 | 1.07 (0.85, 1.35) | 1.00 (0.75, 1.33) | 0.99 (0.74, 1.32) |
|  | Quartile 4 | 240/823 | 0.99 (0.77, 1.26) | 0.90 (0.64, 1.26) | 0.88 (0.63, 1.23) |
|  | *p* for trend |  | 0.764 | 0.744 | 0.677 |

^1^ HOMA-IR = fasting plasma glucose (mmol/l) × fasting insulin (mIU/l) ÷ 22.5. Participants with HOMA-IR ≥2.5 were defined as insulin resistance. ^2^ Model 1, with no adjustment. ^3^ Model 2, adjusted for age, sex (for men and women combined), survey year, BMI, alcohol consumption, smoking status, educational level, occupation, physical activity, menopausal status, grain consumption, fish consumption, seaweed consumption, vegetable consumption, and mushroom consumption. ^4^ Model 3, further adjusted for natural log–transformed blood concentrations of Pb, Hg, or Cd.

**Supplementary Table S5.** Associations between blood Pb, Hg, and Cd concentrations and the TyG index according to sex among participants with HOMA-IR values (3678) ^1^.

|  |  | **Overall** | | | | **Men** | | | | **Women** | | | |
| --- | --- | --- | --- | --- | --- | --- | --- | --- | --- | --- | --- | --- | --- |
|  |  | **Case/total** | **OR (95% CI) ^2^** | **OR (95% CI) ^3^** | **OR (95% CI) ^4^** | **Case/total** | **OR (95% CI) ^2^** | **OR (95% CI) ^3^** | **OR (95% CI) ^4^** | **Case/total** | **OR (95% CI) ^2^** | **OR (95% CI) ^3^** | **OR (95% CI) ^4^** |
| Pb |  |  |  |  |  |  |  |  |  |  |  |  |  |
|  | Continuous | 919/3678 | 3.30 (2.58, 4.20) | 1.48 (1.13, 1.95) | 1.43 (1.08, 1.89) | 606/1672 | 2.69 (1.97, 3.68) | 1.64 (1.13, 2.38) | 1.50 (1.02, 2.20) | 313/2006 | 1.94 (1.36, 2.75) | 1.19 (0.81, 1.74) | 1.22 (0.83, 1.81) |
|  | Quartile 1 | 133/920 | 1.00 (Reference) | 1.00 (Reference) | 1.00 (Reference) | 40/162 | 1.00 (Reference) | 1.00 (Reference) | 1.00 (Reference) | 93/758 | 1.00 (Reference) | 1.00 (Reference) | 1.00 (Reference) |
|  | Quartile 2 | 175/920 | 1.45 (1.09, 1.91) | 0.92 (0.67, 1.27) | 0.90 (0.65, 1.25) | 97/343 | 1.46 (0.90, 2.36) | 0.98 (0.57, 1.69) | 0.95 (0.54, 1.64) | 78/577 | 1.13 (0.78, 1.63) | 0.85 (0.56, 1.30) | 0.86 (0.57, 1.32) |
|  | Quartile 3 | 248/919 | 2.21 (1.67, 2.91) | 1.12 (0.82, 1.53) | 1.09 (0.79, 1.49) | 166/497 | 1.83 (1.14, 2.94) | 1.03 (0.62, 1.72) | 0.97 (0.58, 1.62) | 82/422 | 1.72 (1.16, 2.54) | 1.10 (0.71, 1.72) | 1.11 (0.71, 1.74) |
|  | Quartile 4 | 363/919 | 3.83 (2.93, 5.02) | 1.43 (1.03, 2.00) | 1.37 (0.97, 1.91) | 303/670 | 3.02 (1.94, 4.70) | 1.48 (0.90, 2.44) | 1.34 (0.80, 2.24) | 60/249 | 2.05 (1.34, 3.12) | 1.13 (0.67, 1.90) | 1.15 (0.68, 1.96) |
|  | *p* for trend |  | <.001 | 0.009 | 0.022 |  | <.001 | 0.017 | 0.057 |  | <.001 | 0.537 | 0.485 |
| Hg |  |  |  |  |  |  |  |  |  |  |  |  |  |
|  | Continuous | 919/3678 | 1.75 (1.48, 2.08) | 1.06 (0.87, 1.28) | 1.01 (0.83, 1.23) | 606/1672 | 1.64 (1.33, 2.02) | 1.07 (0.83, 1.37) | 1.02 (0.80, 1.31) | 313/2006 | 1.05 (0.76, 1.46) | 0.92 (0.67, 1.24) | 0.90 (0.65, 1.23) |
|  | Quartile 1 | 163/919 | 1.00 (Reference) | 1.00 (Reference) | 1.00 (Reference) | 62/249 | 1.00 (Reference) | 1.00 (Reference) | 1.00 (Reference) | 101/670 | 1.00 (Reference) | 1.00 (Reference) | 1.00 (Reference) |
|  | Quartile 2 | 211/920 | 1.43 (1.08, 1.91) | 1.24 (0.91, 1.68) | 1.18 (0.87, 1.60) | 122/340 | 2.02 (1.32, 3.08) | 1.66 (1.03, 2.66) | 1.56 (0.97, 2.52) | 89/580 | 0.96 (0.66, 1.40) | 0.99 (0.67, 1.48) | 0.98 (0.66, 1.46) |
|  | Quartile 3 | 220/920 | 1.41 (1.08, 1.84) | 0.97 (0.72, 1.30) | 0.91 (0.67, 1.23) | 161/470 | 1.77 (1.19, 2.61) | 1.17 (0.75, 1.84) | 1.09 (0.70, 1.70) | 59/450 | 0.81 (0.53, 1.22) | 0.80 (0.51, 1.25) | 0.78 (0.50, 1.23) |
|  | Quartile 4 | 325/919 | 2.34 (1.78, 3.09) | 1.10 (0.80, 1.52) | 1.02 (0.74, 1.41) | 261/613 | 2.46 (1.67, 3.63) | 1.22 (0.77, 1.94) | 1.12 (0.71, 1.78) | 64/306 | 1.22 (0.79, 1.88) | 0.99 (0.62, 1.59) | 0.96 (0.59, 1.57) |
|  | *p* for trend |  | <.001 | 0.909 | 0.735 |  | <.001 | 0.857 | 0.606 |  | 0.715 | 0.698 | 0.620 |
| Cd |  |  |  |  |  |  |  |  |  |  |  |  |  |
|  | Continuous | 919/3678 | 1.53 (1.31, 1.79) | 1.23 (0.99, 1.52) | 1.17 (0.94, 1.46) | 606/1672 | 1.86 (1.50, 2.32) | 1.48 (1.10, 1.98) | 1.40 (1.04, 1.89) | 313/2006 | 1.66 (1.29, 2.14) | 0.99 (0.71, 1.37) | 0.98 (0.70, 1.37) |
|  | Quartile 1 | 181/918 | 1.00 (Reference) | 1.00 (Reference) | 1.00 (Reference) | 125/478 | 1.00 (Reference) | 1.00 (Reference) | 1.00 (Reference) | 56/440 | 1.00 (Reference) | 1.00 (Reference) | 1.00 (Reference) |
|  | Quartile 2 | 203/920 | 1.16 (0.89, 1.52) | 0.91 (0.66, 1.24) | 0.87 (0.63, 1.19) | 147/451 | 1.34 (0.97, 1.85) | 1.09 (0.75, 1.59) | 1.05 (0.72, 1.54) | 56/469 | 0.97 (0.60, 1.56) | 0.72 (0.42, 1.23) | 0.70 (0.41, 1.20) |
|  | Quartile 3 | 244/921 | 1.58 (1.23, 2.03) | 1.18 (0.86, 1.62) | 1.13 (0.82, 1.55) | 165/400 | 2.04 (1.45, 2.88) | 1.58 (1.05, 2.37) | 1.51 (1.00, 2.28) | 79/521 | 1.46 (0.95, 2.22) | 0.87 (0.52, 1.45) | 0.85 (0.51, 1.41) |
|  | Quartile 4 | 291/919 | 1.89 (1.48, 2.43) | 1.34 (0.95, 1.89) | 1.25 (0.88, 1.76) | 169/343 | 2.83 (2.04, 3.93) | 2.04 (1.30, 3.22) | 1.88 (1.19, 2.99) | 122/576 | 1.90 (1.26, 2.85) | 0.82 (0.48, 1.40) | 0.80 (0.47, 1.38) |
|  | *p* for trend |  | <.001 | 0.049 | 0.112 |  | <.001 | 0.001 | 0.004 |  | <.001 | 0.677 | 0.639 |

Pb, lead; Hg, mercury; Cd, cadmium; TyG index, triglyceride glucose index; OR, odds ratio; CI, confidence interval.

^1^ The cut-off point was 8.828, the highest quartile of the TyG index among 3,678 participants with HOMA-IR values.

^2^ Model 1, with no adjustment.

^3^ Model 2, adjusted for age, sex (for men and women combined), survey year, BMI, alcohol consumption, smoking status, educational level, occupation, physical activity, menopausal status, grain consumption, fish consumption, seaweed consumption, vegetable consumption, and mushroom consumption.

^4^ Model 3, further adjusted for natural log–transformed blood concentrations of Pb, Hg, or Cd.

**Supplementary Table S6.** Comparing current study with Moon SS’s study.

|  |  | **Moon SS’ study** | **Our study** |
| --- | --- | --- | --- |
| Population | |  |  |
|  | Survey year | 2009–2010 (2 years) | 2005, 2008–2013, 2016 (8 years) |
|  | Total sample size | 3184 | 9645 |
|  | Population | Participants aged 30 years and older | Participants aged 19 years and older |
|  | Disease history exclusion | - | Type 2 diabetes, hyperlipidemia, hypertension, stroke, myocardial infarction, and cancer |
|  | Missing value exclusion | - | Covariates of BMI, alcohol consumption, smoking status, educational level, occupation, physical activity, menopausal status, and dietary consumptions of grain, fish, seaweed, vegetables, and mushrooms |
| TyG index | |  |  |
|  | Survey year | 2009–2010 (2 years) | 2005, 2008–2013, 2016 (8 years) |
|  | Sample size analyzed | 3184 | 9645 |
|  | Geometric means | 8.64 (8.61–8.67) | 8.43 (8.41–8.44) |
|  | Prevalence | Cut-off points referred from:  1) Iranian study: 876 (27.5%)  2) Japanese study: 2241 (70.4%)  3) Chinese study: 1317 (41.4%)  4) Korean study: 1799 (56.5%) | Cut-off points referred from:  1) Iranian study: 1709 (17.7%)  2) Japanese study: 5564 (57.7%)  3) Chinese study: 2755 (28.6%)  4) Korean study:4077 (42.3%) |
| HOMA-IR | |  |  |
|  | Survey year | 2009–2010 (2 years) | 2008–2010 (3 years) |
|  | Sample size analyzed | 3184 | 3678 |
|  | Geometric means | 2.25 (2.20–2.30) | 2.06 (2.02–2.10) |
|  | Prevalence | 1162 (36.5%) | 1047 (28.5%) |

**Supplementary Table S7.** The limits of detection (LOD) for blood Pb, Hg, and Cd ^1^.

| Year | Pb (μg/dL) | Hg (μg/L) | Cd (μg/L) |
| --- | --- | --- | --- |
| 2005 | 0.230 | 0.360 | 0.300 |
| 2008 | 0.120 | 0.158 | 0.056 |
| 2009 | 0.142 | 0.172 | 0.087 |
| 2010 | 0.142 | 0.170 | 0.062 |
| 2011 | 0.148 | 0.175 | 0.081 |
| 2012 | 0.189 | 0.200 | 0.098 |
| 2013 | 0.178 | 0.162 | 0.088 |
| 2016 | 0.139 | 0.163 | 0.084 |

^1^ The limits of detection (LOD) values for 2008–2013, and 2016 were derived from the annual reports of Clinical Laboratory Test for the Korea National Health and Nutrition Examination Survey, while the 2005 values were referred to an experimental article (Kim NS, et al. 2011) due to lack of information in the annual report.
